# Electroacupuncture improves repeated social defeat stress-elicited social avoidance and anxiety-like behaviors by reducing Lipocalin-2 in the hippocampus

**DOI:** 10.1186/s13041-021-00860-0

**Published:** 2021-09-26

**Authors:** Yi-Hung Chen, Sheng-Yun Xie, Chao-Wei Chen, Dah-Yuu Lu

**Affiliations:** 1 Graduate Institute of Acupuncture Science, China Medical University, Taichung, Taiwan; 2 Department of Pharmacology, School of Medicine, China Medical University, Taichung, Taiwan; 3 Institute of New Drug Development, China Medical University, Taichung, Taiwan; 4 Department of Photonics and Communication Engineering, Asia University, Taichung, Taiwan

**Keywords:** Post-traumatic stress disorder, Electroacupuncture, Next-generation sequencing, Lipocalin-2, Intracerebroventricular injection, Repeated social defeat stress

## Abstract

**Background:**

Post-traumatic stress disorder (PTSD) is a trauma-related disorder that is associated with pro-inflammatory activation and neurobiological impairments in the brain and leads to a series of affective-like behaviors. Electroacupuncture (EA) has been proposed as a clinically useful therapy for several brain diseases. However, the potential role of EA treatment in PTSD and its molecular and cellular mechanisms has rarely been investigated.

**Methods:**

We used an established preclinical social defeat stress mouse model to study whether EA treatment modulates PTSD-like symptoms and understand its underlying mechanisms. To this end, male C57BL/6 mice were subjected to repeated social defeat stress (RSDS) for 6 consecutive days to induce symptoms of PTSD and treated with EA at Baihui (GV 20) and Dazhui (GV 14) acupoints.

**Results:**

The stimulation of EA, but not needle insertion at Baihui (GV 20) and Dazhui (GV 14) acupoints effectively improved PTSD-like behaviors such as, social avoidance and anxiety-like behaviors. However, EA stimulation at the bilateral Tianzong (SI11) acupoints did not affect the PTSD-like behaviors obtained by RSDS. EA stimulation also markedly inhibited astrocyte activation in both the dorsal and ventral hippocampi of RSDS-treated mice. Using next-generation sequencing analysis, our results showed that EA stimulation attenuated RSDS-enhanced lipocalin 2 expression in the hippocampus. Importantly, using double-staining immunofluorescence, we observed that the increased lipocalin 2 expression in astrocytes by RSDS was also reduced by EA stimulation. In addition, intracerebroventricular injection of mouse recombinant lipocalin 2 protein in the lateral ventricles provoked social avoidance, anxiety-like behaviors, and the activation of astrocytes in the hippocampus. Interestingly, the overexpression of lipocalin 2 in the brain also altered the expression of stress-related genes, including monoamine oxidase A, monoamine oxidase B, mineralocorticoid receptor, and glucocorticoid receptor in the hippocampus.

**Conclusions:**

This study suggests that the treatment of EA at Baihui (GV 20) and Dazhui (GV 14) acupoints improves RSDS-induced social avoidance, anxiety-like behaviors, astrocyte activation, and lipocalin 2 expression. Furthermore, our findings also indicate that lipocalin 2 expression in the brain may be an important biomarker for the development of PTSD-related symptoms.

**Supplementary Information:**

The online version contains supplementary material available at 10.1186/s13041-021-00860-0.

## Background

Post-traumatic stress disorder (PTSD), a common consequence of trauma, is a trauma and stressor-related disorder caused by exposure to severe traumatic events [1]. The clinical hallmarks of PTSD include avoidance of trauma reminders, re-experiencing a traumatic event, arousal, and hyperarousal symptoms. PTSD is an anxiety disorder that may persist for decades if not treated [2]. Repeated environmental stress puts patients at high risk for various psychiatric disorders that alter the morphology and activity of neurons in the central nervous system (CNS) [3] and induces maladaptive emotional and cognitive behaviors [4]. Social stress is a common environmental stress in human society that increases the risk of psychopathologies [5]. Repeated social defeat stress (RSDS) is a psychosocial stress animal model that induces symptoms of PTSD, such as emotional change and anxiety-like behavior [6, 7]. Importantly, experimental animals undergone repeatedly social defeat were exhibited social avoidance of the stressors [8]. RSDS was also reported to enhance vulnerability to subsequent adversity, which promotes anxiety recurrence of stress sensitization [9]. Our recent studies also demonstrated that mice subjected to RSDS led to symptoms of PTSD, including social avoidance and anxiety-like behaviors [10, 11].

Accumulating evidence has shown that psychological stress causes neuroinflammation and immune responses, which are important factors in the pathogenesis of psychiatric illnesses [12]. In clinical studies, the activation of the immune system contributes to chronic stress responses and is implicated in the development of mood disorders [13]. Rodents exposed to various environmental stressors increase circulating pro-inflammatory cytokines, which is consistent with clinical observations [14]. Social defeat has also been reported to affect proinflammatory immune processes, including variations in pro-inflammatory cytokines, such as interleukin (IL)-1β, IL-6, and tumor necrosis factor (TNF)-α in the brain [15]. RSDS initiates the release of inflammatory Ly6Chigh monocytes and Ly-6c intermediate granulocytes into circulation, and these monocytes are characterized by glucocorticoid insensitivity [16]. Notably, RSDS also promotes the accumulation of peripheral monocytes in the brain and causes prolonged anxiety-like behavior for one more week [17]. In addition, using green fluorescent protein (GFP)-expressing bone marrow chimeras indicated that RSDS promoted the recruitment of peripheral macrophages in the hippocampus [18]. The previous finding also showed that RSDS increased the number of recruited monocytes that enhanced the inflammatory response and was required for the induction of anxiety-like behavior [19].

There are at least two techniques used in acupuncture therapy: manual acupuncture (MA) and electroacupuncture (EA), a modified form of traditional manual acupuncture. A combines the therapeutic effects of transcutaneous electric nerve stimulation and MA, which can be standardized by frequency, current, waveform, and length [20]. Previous reports have shown that MA at Neiguan (PC6) improves acute restraint stress-induced anxiety in rats [21]. Clinically, low-frequency EA suppresses focal epilepsy and improves epilepsy-induced sleep disruption [22]. Importantly, increasing clinical studies have tested the efficacy of acupuncture on PTSD. A recent study reported the use of acupuncture intervention to reduce earthquake-related pain and psychological symptoms in affected individuals of earthquake-stricken areas of central Italy [23]. Surprisingly, a clinical trial observed that acupuncture exerts large treatment effects in patients with PTSD and maintains the reduction of symptoms for 3 months [24]. Further, a systemic review of clinical studies with meta-analysis found that acupuncture was effective for PTSD patients [25]. In addition, another systematic review and meta-analysis of acupuncture also suggests that acupuncture treatment in adults with PTSD is favored at post-intervention for depressive symptoms, anxiety symptoms, and sleep quality [26]. Furthermore, a variety of regulatory mechanisms have been proposed that act in improvement of EA stimulation in PTSD animal models [27]. EA reduces the anxiety symptoms by down regulating the hypothalamic–pituitary–adrenal (HPA) axis [28], upregulating hippocampal thioredoxin expression [29], increasing expression of BDNF, and TrkB signaling in amygdala [30], increasing expression of cannabinoid receptor in prefrontal cortex [31], and preventing hippocampal neurogenesis [32]. It has also been reported that EA treatment improves the sleep disturbance by regulating cytokine expression in hippocampus [33].

Chronic and repeated stress may facilitate the onset and exacerbate the symptoms of various psychiatric disorders, such as PTSD. We recently investigated the molecular mechanism of individual susceptibility to environmental stress, which led to unexpected discoveries in biomarkers after stress [11]. In this study, we used a preclinical mouse model of RSDS to elaborate the clinical symptoms of PTSD under social stress in the human society. Although many studies have reported the beneficial effects of acupuncture in many CNS disorders, the precise mechanism remains unknown. The present study elucidates the therapeutic effects of EA on RSDS-induced behavioral impairment. Our results also identified biomarkers of RSDS and developed EA treatments for stress-related mood disorders.

## Materials and methods

### Animals, RSDS, and experimental design

Eight-week-old male C57BL/6 mice were obtained from the National Laboratory Animal Center (Taipei, Taiwan). Under standard laboratory conditions (21 ± 2 °C, 12-h light/dark cycle, with food and water available ad libitum), the mice were housed in groups before and after the social defeat experiments and during behavioral testing. A total of 13 25-week-old CD-1 (ICR) male mice (National Laboratory Animal Center) were used as aggressors in the social defeat paradigm.

All animal experiments were performed in accordance with the Animal Care and Use Guidelines of China Medical University (IACUC Approval No. CMUIACUC-2019-139). The social defeat stress paradigm was performed according to a standard social defeat stress protocol [8] but with some modifications. The aggressor CD-1 mice were screened based on their aggressiveness to an experimental C57BL/6 mouse, as measured by the latency (must be < 60 s) and the number of attacks (must attack in at least two consecutive sessions) during the observation period (180 s). RSDS was applied between 15:00 and 17:00 in a sound-attenuated room under dim light. The experimental C57BL/6 mice were subjected to a new CD-1 aggressor mouse (one CD-1 aggressor mouse attacked three C57BL/6 mice in each CD-1 mouse cage) for 2 h once daily for 6 consecutive days. After 2 h of physical contact, the experimental mice were separated and housed in their home cages for the next 22 h. The social interaction, light–dark box, or elevated plus maze (EPM) tests were performed 24 h after the last defeat episode. For tissue collection after the behavioral test, the mice were euthanized, and the brain tissues were dissected and stored at − 80 °C.

### Next generation sequencing (NGS)

In each group (Control, RSDS, and RSDS + EA group), 13 hippocampal tissues were pooled together as one group and sent for RNA sequencing. After the behavioral tests, the mice were sacrificed, and the hippocampal tissue was removed quickly and put in liquid nitrogen for quick freezing. The hippocampal tissue was stored at −80 °C until tissue processing. Total RNA was extracted from the tissue using TRIzol reagent (Sigma-Aldrich, St. Louis, MO, USA). For RNA isolation, purified RNA was quantified at OD260 nm using an ND-1000 spectrophotometer (Nanodrop Technology, USA) and quantified using Bioanalyzer 2100 (Agilent Technology, USA) with an RNA 6000 LabChip kit (Agilent Technology, USA). The DNA libraries were used for cluster generation and sequencing using cBot Operation for HD V2.5 Reagent and HiSeq X Operation for HD v2.5 reagent v1.3 (Illumina). The sequence was determined using Illumina's sequencing-by-synthesis technology (Illumina, USA), and the sequencing data (FASTQ reads) were generated using Welgene Biotech's pipeline based on Illumina's base calling program bcl2fastq v2.20. Genes with *p* values ≤ 0.05, and ≥ twofold changes were considered to be significantly differentially expressed [34, 35]. Genes with low expression levels (< 0.3 FPKM value) in these three groups were excluded. In addition, GeneMANIA5 was employed to generate protein-interaction network [36].

### Needle insertion and EA stimulation

EA treatment in animal model was performed as described in our previous report [37]. Briefly, to evaluate the effects of needle insertion or EA, C57BL/6 mice were individually acclimated in rectangular observation boxes and anesthetized with 2% isoflurane. Under anesthesia via nose mask, the mice were arranged for needle insertion or EA stimulation. A pair of stainless-steel acupuncture needles were inserted into the murine equivalent of the acupoints, Dazhui (GV14) and Baihui (GV20), for 20 min once a day for 5 successive days. Meanwhile, the control mice were only anesthetized with isoflurane for 20 min. The acupoint “Baihui (GV20, Governing vessel meridian 20)” is located at the vertex of the dorsal midline. The acupoint “Dazhui (GV14, Governing vessel meridian 14),” is located at the depression between the spinous processes of the seventh cervical and the first thoracic vertebrae on the dorsal midline. To accomplish this, acupuncture needles (Beijing Zhongyan Taihe Medical Instrument Co., Ltd.; 0.16 × 7 mm) were inserted to a depth of approximately 3 mm, after which they were stimulated at a frequency of 2 Hz and an intensity of 1 mA for 20 min with a pulse width of 150 μs, using an EA Trio 300 stimulator (Ito, Japan). The acupoint Tianzong (SI11, small intestine meridian 11), is located in the region of the scapula, in the depression at the center of the subscapular fossa [38]. A sham EA was performed by bilateral insertion of a pair of stainless-steel acupuncture needles into the middle of each scapula, which is equivalent to the Tianzong (SI11).

### Preparation of behavioral tests

The social interaction, light–dark box, and EPM tests were performed 24 h after the last defeat episode. The mice were acclimatized to a separate room for at least 1 h prior to each test, and all experiments were performed under low light conditions. Between each test, the apparatus was cleaned with 75% ethanol. In addition, the light–dark box test was conducted at the first task, social interaction test was conducted at the second task, EPM test was conducted at the end, and each behavioral test was conducted within an interval of more than 2 h.

### Behavioral tests I: social interaction test

The social interaction test was performed as described in our previous studies [10, 11]. Briefly, at the end of the social defeat paradigm, the experimental C57BL/6 mice were allowed to freely explore on an open field arena (53 cm × 53 cm × 38 cm) towards an unfamiliar target mice in a mesh cage (10 cm × 7 cm × 38 cm) and was measured in 5 min. In the first 2.5-min session (target mice absent), the C57BL/6 mouse was introduced to the open field chamber with an empty mesh cage located at one end of the field. In the second 2.5-min session (target mice present), the mesh cage contained an unfamiliar CD-1 mice. The area (24 cm × 14 cm) surrounding the mesh cage was defined as the interaction zone. The tract of the C57BL/6 mice, duration of the measurement period, and time spent in the interaction or corner zones (8 cm × 8 cm) were measured using the Noldus EthoVision XT 12 behavioral tracking system (Noldus Information Technology, Wageningen, Netherlands). The social interaction ratio was obtained by dividing the time spent in the interaction zone when the target was present by the time spent in the interaction zone when the target was absent.

### Behavioral tests II: light–dark box test

The light–dark box test uses a Plexiglas box (45 cm × 20 cm × 29 cm) divided into dark and light compartments separated by an open door (diameter, 10 cm) located in the center of the partition at the floor level. At the start of the test, each mouse was placed in a dark chamber and the door was opened. Mice were allowed to freely explore the apparatus for 5 min and the Noldus EthoVision XT 12 behavioral tracking system (Noldus Information Technology, Wageningen, Netherlands). The time spent in the light and dark compartments was measured and analyzed.

### Behavioral tests III: EPM test

The EPM test was performed according to our previous studies [10, 11]. Briefly, the EPM apparatus was made from white Plexiglas with a white floor and consisted of two opposite open arms (30 cm × 5 cm × 39.5 cm) and two enclosed arms (30 cm × 5 cm × 39.5 cm) surrounded by a 25.5 cm-high white wall. These arms were connected by a central area (5 cm × 5 cm), and the device was elevated 50 cm above the floor. At the start of the test, each mouse was placed at the end of the open arms facing the center of the maze. Mice were allowed to freely explore the apparatus for 5 min and the Noldus EthoVision XT 12 behavioral tracking system (Noldus Information Technology, Wageningen, Netherlands) was used to measure the time spent in the open and closed arms.

### Immunohistochemistry

The dissected tissue was fixed in 10% neutral buffered formalin overnight and dehydrated in 30% sucrose solution for 2 days at 4 °C. The dissected tissue was placed on a tissue base mold and the entire tissue was covered with an FSC 22 frozen section compound (3801480; Leica Surgipath, Buffalo Grove, USA). The frozen tissues were sectioned to a thickness of 30 µm using a cryotome, and placed on glass slides suitable for immunohistochemistry and immunofluorescence staining. Coronal slices on glass slides were quenched for endogenous peroxidases with 3% hydrogen peroxide, and blocked by incubation in 1% Triton X-100 and 5% bovine serum albumin for 1 h The anti-glial fibrillary acidic protein (GFAP) primary antibody (Z0334, Agilent Dako, USA) was applied to the slides at a dilution of 1:50, followed by overnight incubation at 4 °C. After several washes, the sections were incubated with the secondary antibody and HRP-conjugated streptavidin, and developed with diaminobenzene reagent for 5 min. Bound antibodies were detected using an immunoperoxidase secondary detection system (Merck KGaA, Darmstadt, Germany), and the slices were then washed and mounted on glass slides with mounting medium (Leica Surgipath, Buffalo Grove, USA).

### Immunofluorescence staining

In the double-labeling immunofluorescence assay, the brain slices were incubated with rabbit anti–lipocalin 2 antibody (bs-1373R, Bioss Antibodies, Woburn, Massachusetts, USA) and rat anti-GFAP (2.2B10, Invitrogen, Waltham, Massachusetts, USA) overnight at 4 °C. After washing, the sections were incubated for 1 h with goat anti-rat IgG (H + L) Alexa Fluor 594 secondary antibodies (1:200; Invitrogen, Waltham, Massachusetts, USA) and goat anti-rabbit IgG Alexa Fluor 488 secondary antibodies (1:200; Invitrogen, Waltham, Massachusetts, USA) for 1 h, and incubated for 3 min with DAPI (0.5 mg/mL at a dilution of 1:1000) counterstaining, and mounted on glass slides with mounting medium. The images were captured and analyzed by confocal microscopy (ANDOR Dragonfly high-speed confocal system, ANDOR, Dragonfly 200) with 20× and 100× objectives.

### Reverse transcription and real-time polymerase chain reaction

Real-time polymerase chain reaction (PCR) was performed to measure the expression levels of mineralocorticoid receptor (MR), glucocorticoid receptor (GR), monoamine oxidase (MAO)-A, MAO-B, lipocalin 2, and β-actin. Total RNA was isolated with TRIzol (Sigma-Aldrich, St. Louis, MO, USA), and the concentration of RNA was measured using a BioDrop spectrophotometer. The RT reactions were performed with 2 μg of total RNA, which was reverse transcribed into cDNA. Target gene expression was detected using quantitative real-time PCR. PCR was performed using SYBR Green Master Mix (Applied Biosystems, Foster City, CA, USA) on a StepOne Plus Real-Time PCR System. The data were analyzed by comparing the cycle threshold (Ct) values of the different groups using the method of 2^−ΔΔCt^. The oligonucleotide primers were MR: 5- GAAGAGCCCCTCTGTTTGCAG -3 and 5- TCCTTGAGTGATGGGACTGTG -3; GR: 5- AGCTCCCCCTGGTAGAGAC -3 and 5- GGTGAAGACGCAGAAACCTTG -3; MAO-A: 5- AACGACTTGCTAAACTACAT -3 and 5- AGCAGAGAAGAGCCACAGAA -3; MAO-B: 5- GAAAACTGGTACGTCTCACC -3 and 5- GAGCTGTTGCTGACAAGATG -3; lipocalin 2: 5- CCCCATCTCTGCTCACTGTC -3 and 5- TTTTTCTGGACCGCATTG -3; β-actin: 5- AGAGCTACGAGCTGCCTGAC -3 and 5- AGCACTGTGTTGGCGTACAG -3.

### Western blotting

Tissue samples obtained from the hippocampus of the mice were homogenized in radioimmunoprecipitation lysis buffer containing protease and phosphatase inhibitor cocktails. Protein samples (50 μg) were denatured in Laemmli buffer at 95 °C for 5 min, separated by sodium dodecyl sulfate–polyacrylamide gel electrophoresis with a 15% Tris–glycine gel electrophoresis, and then transferred on PVDF membranes (Millipore, Bedford, MA, USA). Membranes were blocked with non-fat dry milk (5%) for 1 h. and then incubated overnight at 4 °C with the following primary antibodies: anti-NGAL (sc-515876, Santa Cruz Biotechnology, Inc., Dallas, TX, USA) and GAPDH (G8795, Sigma-Aldrich, St. Louis, MO, USA). After washing with TBST buffer, the membranes were incubated with anti-mouse or anti-rabbit HRP-conjugated secondary antibodies. Protein bands were visualized using enhanced chemiluminescence and Kodak X-OMAT LS film (Eastman Kodak, Rochester, NY, USA). The data were quantified using an ImageJ software (National Institutes of Health, Bethesda, MD, USA).

### Intracerebroventricular injection

The mice were anesthetized and positioned on a stereotaxic instrument, and the i.c.v. administration of 7 ng mouse recombinant lipocalin 2 protein (1857-LC-050, Biotechne) 1 μg/mL in the lateral ventricles using a 10-µL Hamilton syringe with a 26S-gauge needle (Hamilton, Reno, NV, USA) mounted to an automated microinjector (Holliston, MA, USA). The infusions were made at a rate of 0.5 μL /min for 10 min. The i.c.v. injection was located at −0.8 mm with respect to bregma, 1.5 mm to the right from the center, and −2.0 mm in depth (Fig. 8A). At the end of the injection, the needle was left in place for an additional 10 min before being retracted by slowly withdrawing the syringe to prevent any reflux. The skull was then cleaned, and the incision was sutured.

### Statistical analysis

The values were determined using GraphPad Prism 8 software (GraphPad Software, Inc., La Jolla, CA, USA). The results are presented as the mean ± standard error of mean. Statistical analysis between two samples was performed using the Student’s t-test. For multiple comparisons, one-way analysis of variance (ANOVA) was performed, followed by the Bonferroni or Tukey tests. In all cases, a *p*-value < 0.05, was considered to be statistically significant. The *p*-values are indicated in the "Results" and "Figure legends" sections. No pretest was used to determine the sample size.

## Results

### EA stimulation at the Baihui (GV 20) and Dazhui (GV 14) acupoints improves RSDS-induced social avoidance

**Fig. 1:**
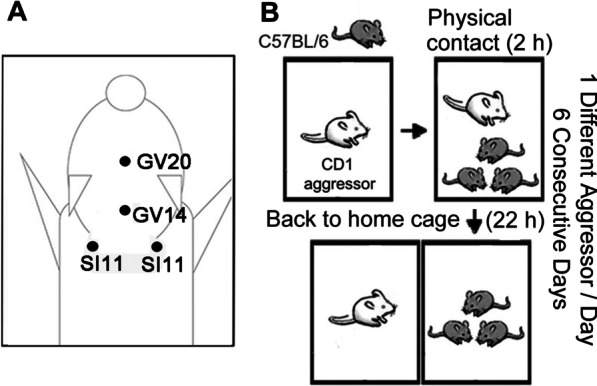
Schematic diagrams of the experimental protocols. C57BL/6 mice were individually anesthetized via a nose mask. A pair of stainless-steel acupuncture needles were inserted into the murine equivalent of the acupoints: Dazhui (GV14) and Baihui (GV20), or bilateral Tianzong (SI11) (**A**). **B** The procedures of RSDS paradigm

**Fig. 2:**
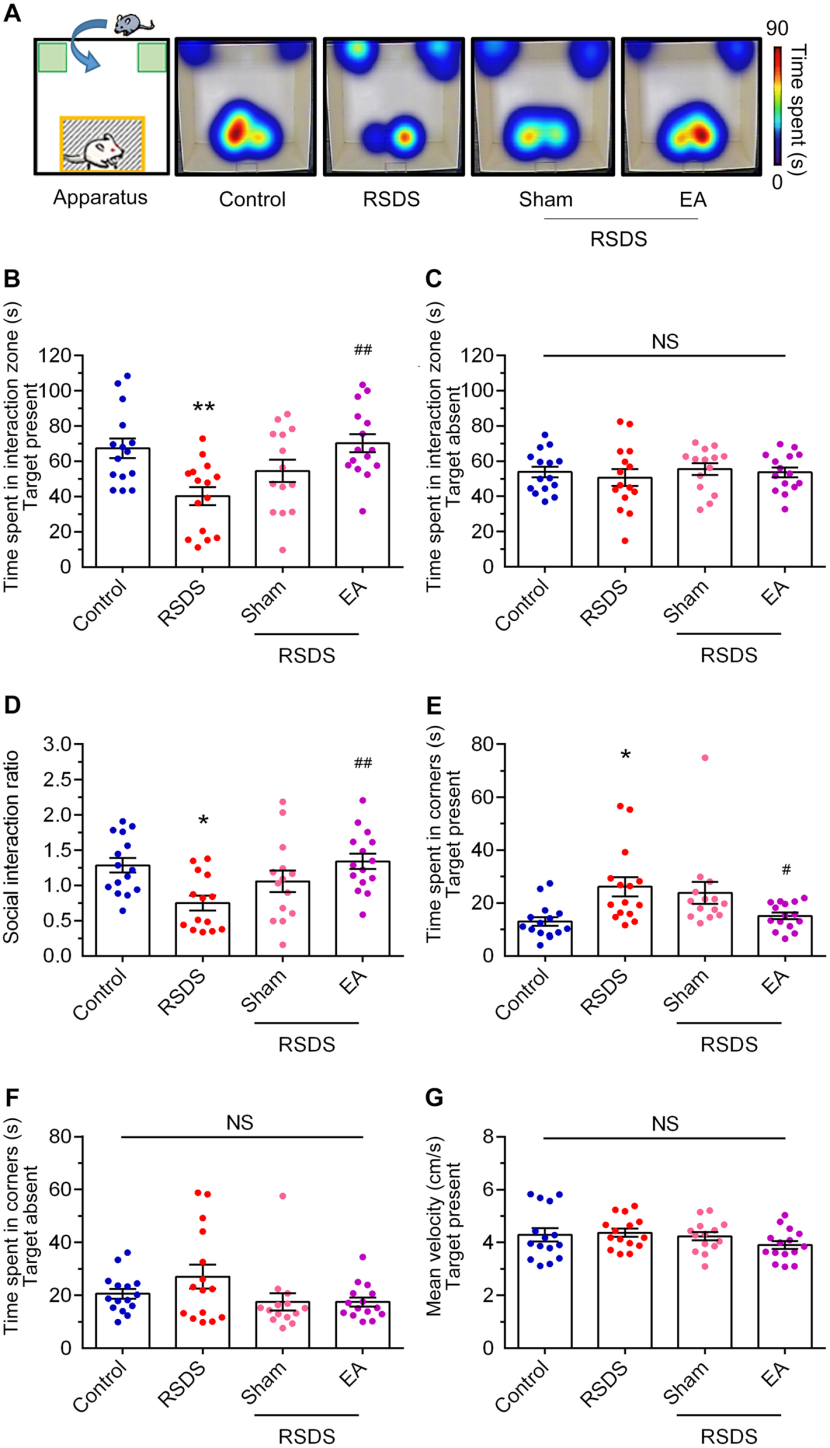
Electroacupuncture stimulation at the Baihui (GV 20) and Dazhui (GV 14) acupoints improves RSDS-evoked social avoidance behavior. **A** Representative heat maps for the social activities of each group. After RSDS, the experimental mice were introduced to the social apparatus (Interaction zone: grey area; corner zones: green areas). The time spent in the interaction zone was determined when the target was present (**B**) or absent (**C**). **D** The social interaction ratio was calculated by dividing the time spent in the interaction zone while the target was present by the time spent in interaction zone while the target was absent. The time spent in corners were determined by the interaction zone with target (**E**) or without target (**F**). **G** The velocity traveled with the target present during the test. Quantitative data are presented as the mean ± SEM (control: n = 15; RSDS: n = 15; RSDS/Sham: n = 14; RSDS/EA n = 15). One-way ANOVA with a post-hoc Tukey test was used to examine the significance of the mean. * *p* < 0.05 vs. the control group. ** *p* < 0.01 vs. the control group. # *p* < 0.05 vs. the RSDS group. ## *p* < 0.01 vs. the RSDS group. NS: not significant

C57BL/6 mice were individually anesthetized via a nose mask, and treated with EA at the following acupoints: Dazhui (GV14) and Baihui (GV20), or bilateral Tianzong (SI11) (Fig. 1A). The RSDS paradigm was shown in Fig. 1B. First, to measure the effect of EA treatment in RSDS-treated mice, we determined the behaviors of EA-stimulated mice at the Baihui and Dazhui acupoints (GV20 and GV14) or by needle insertion (sham group). Figure 2A shows the representative heat maps of the social interaction analysis following RSDS. RSDS mice treated with sham stimulation, like the RSDS group, spent less time interacting when a target was present compared with the control group. RSDS mice treated with EA stimulation spent more time interacting when a target was present, compared with the RSDS group. In addition, RSDS mice treated with EA spent a similar amount of time in the interaction zone with the target present, similar to the control group (Fig. 2B). There was no significant difference in the time spent in the interaction zone between the sham and RSDS groups (Fig. 2B, RSDS vs. control, *p* < 0.01; RSDS + sham vs. RSDS, NS; RSDS + EA vs. RSDS, *p* < 0.01; one-way ANOVA). As shown in Fig. 2C, there was no significant difference in the time spent in the interaction zone with the target mice absent in each group. Similarly, the analysis of the social interaction ratio of the RSDS mice was improved by EA treatment but not by sham treatment (Fig. 2D; RSDS vs. control, *p* < 0.05; RSDS + sham vs. RSDS, NS; RSDS + EA vs. RSDS, *p* < 0.01; one-way ANOVA). In contrast, the time spent in the corner zones of RSDS was also significantly reduced by EA stimulation but not by sham treatment (Fig. 2E; RSDS vs. control, *p* < 0.05; RSDS + sham vs. RSDS, NS; RSDS + EA vs. RSDS, *p* < 0.05; one-way ANOVA). There was no significant difference in the time spent in the corner zone with the target mice absent between the groups (Fig. 2F). In addition, movement velocity was not significantly different between the groups (Fig. 2G). Collectively, these data indicate that EA stimulation at the Baihui and Dazhui acupoints improves RSDS-induced social avoidance and exerts potential therapeutic effects compared to needle insertion.

### EA stimulation at the Baihui (GV 20) and Dazhui (GV 14) acupoints improves RSDS-elicited anxiety-like behaviors

**Fig. 3:**
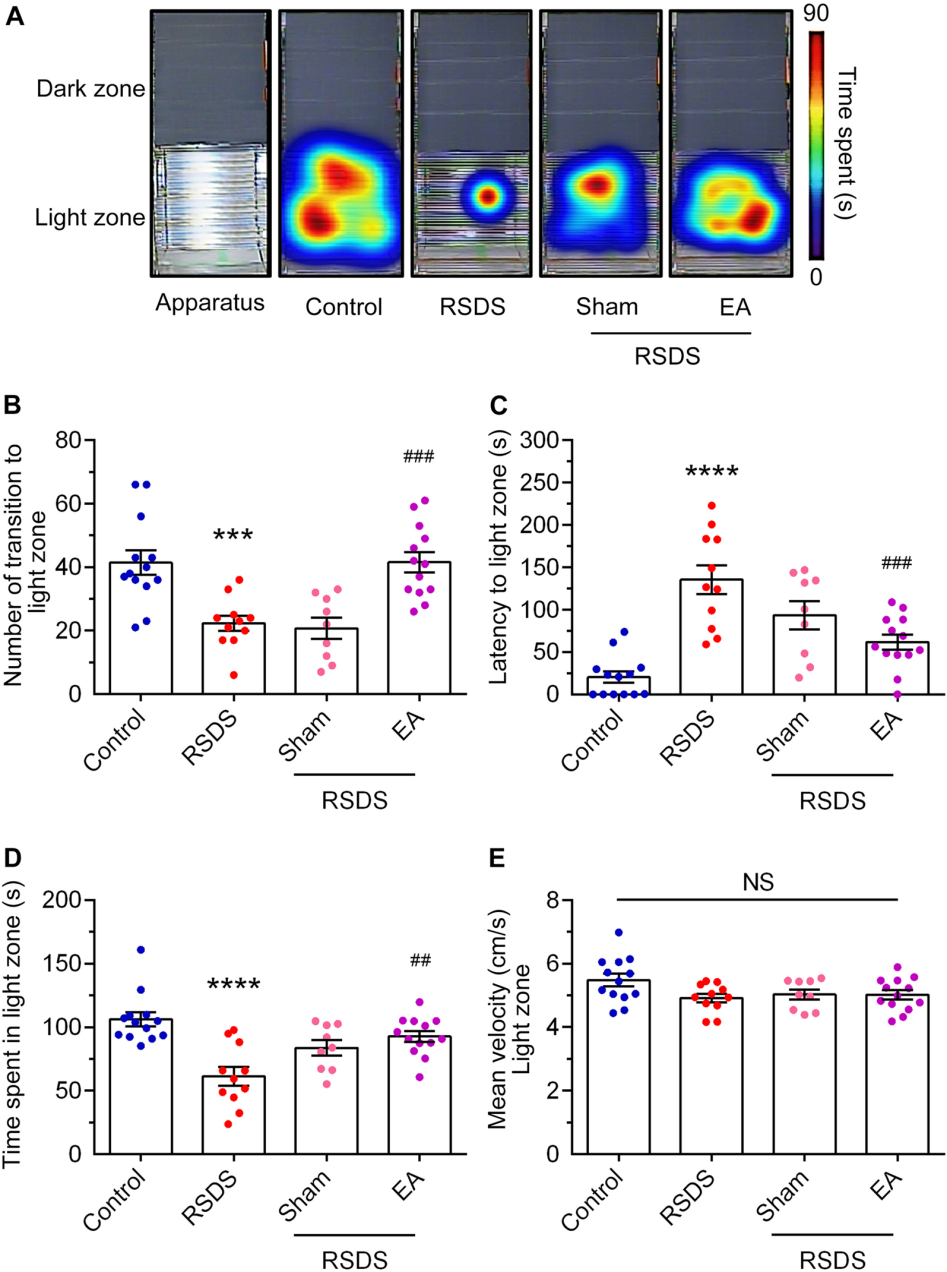
EA stimulation at the Baihui (GV 20) and Dazhui (GV 14) acupoints improves RSDS-evoked anxiety-like behaviors in the light–dark box task. **A** Representative heat maps for the activities of each group during the light–dark test. The frequency spent in the light compartment (**B**), the latency to the first entry into light compartment (**C**), or the cumulative time in the light compartment (**D**) were determined. The velocity traveled in the light compartment was measured in (**E**). Quantitative data are presented as the mean ± SEM (control: n = 13; RSDS: n = 11; RSDS/Sham: n = 9; RSDS/EA n = 13). One-way ANOVA with a post-hoc Tukey test was used to examine the significance of the mean. *** *p* < 0.001 vs. the control group. **** *p* < 0.0001 vs. the control group. ## *p* < 0.01 vs. the RSDS group. ### *p* < 0.001 vs. the RSDS group. ANOVA: analysis of variance, EA: electroacupuncture, NS: not significant, RSDS: repeated social defeat stress, SEM: standard error of mean

The affective-like activities of RSDS mice treated with EA were determined. Light and dark (L/D) box task has been widely used to examine anxious behavior in rodent models [39]. Figure 3A shows the representative heat maps of the light compartment in the L/D box following RSDS. The RSDS group spent less time traveling in the light compartment than the control group. The time spent traveling in the light compartment was significantly increased in the group of RSDS mice treated with EA, compared with the sham group (Fig. 3B). In addition, both the RSDS and RSDS groups with sham treatment spent a similar amount of frequency traveling in the light compartment (Fig. 3B). The mice in the RSDS group with EA treatment traveled more frequently in the light compartment than those in the RSDS group. (RSDS vs. control, *p* < 0.001; RSDS + sham vs. RSDS, NS; RSDS + EA vs. RSDS, *p* < 0.001; one-way ANOVA). The latency to the first entry into the light compartment of the RSDS group was markedly increased compared to that of the control group (Fig. 3C). Surprisingly, the latency to the first entry into the light compartment was also decreased by EA stimulation but not by sham treatment (Fig. 3C; RSDS vs. control, *p* < 0.0001; RSDS + sham vs. RSDS, NS; RSDS + EA vs. RSDS, *p* < 0.001; one-way ANOVA). Moreover, the cumulative time spent in the light compartment of the group of RSDS mice treated with EA was significantly increased compared to that in the RSDS group (Fig. 3D; RSDS vs. control, *p* < 0.0001; RSDS + sham vs. RSDS, NS; RSDS + EA vs. RSDS, *p* < 0.01; one-way ANOVA). In addition, there was no significant difference in the movement velocity between the groups (Fig. 3E).

**Fig. 4:**
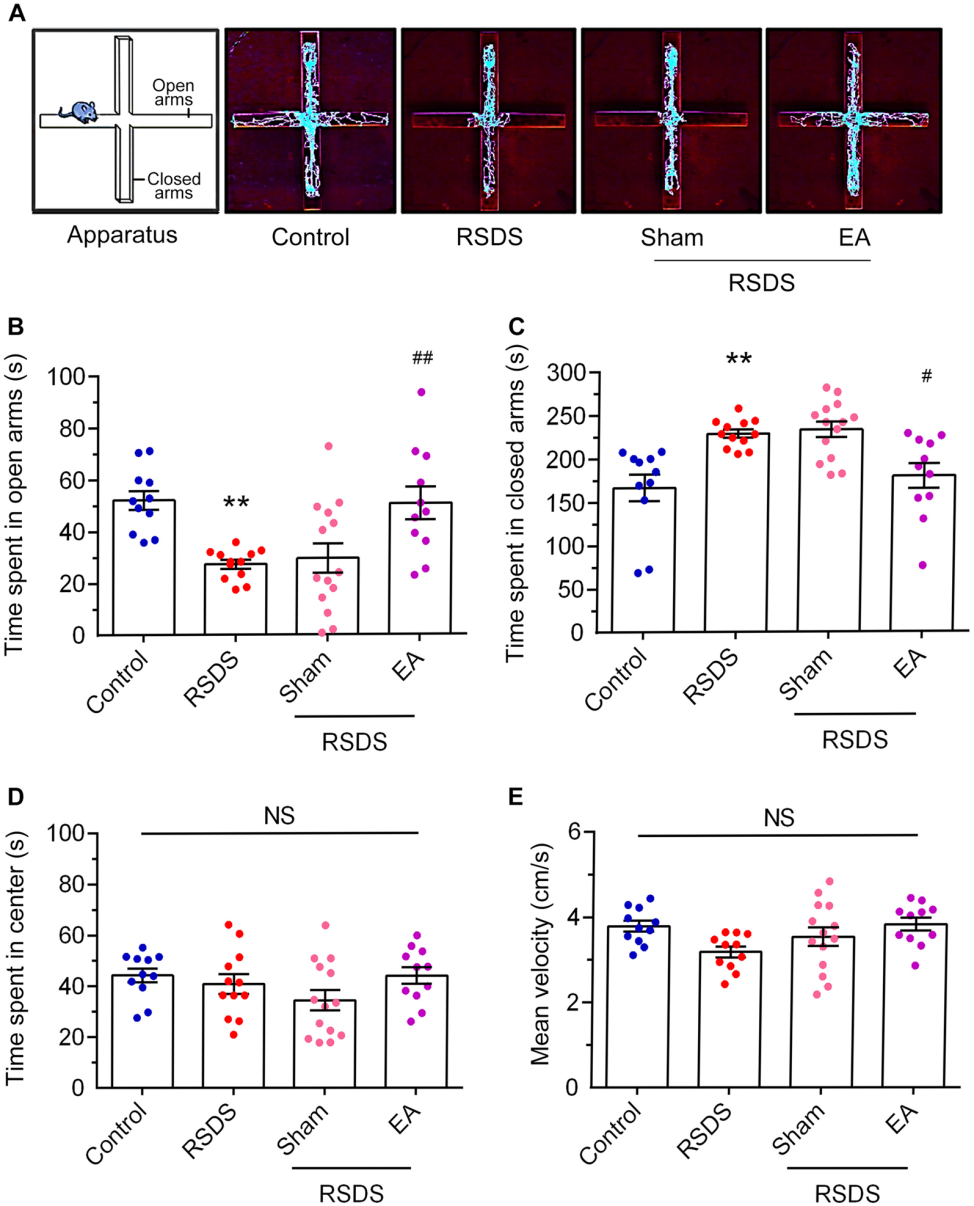
EA stimulation at the Baihui (GV 20) and Dazhui (GV 14) acupoints improves RSDS-evoked anxiety-like behaviors in EPM task. (**A**) Representative traces for the activity on EPM apparatus for each group. The time spent in open arms (**B**), closed arms (**C**), or center (**D**) of the EPM apparatus were determined. The velocity traveled on the EPM apparatus was measured in (**E**). Quantitative data are presented as the mean ± SEM (control: n = 11; RSDS: n = 12; RSDS/Sham: n = 14; RSDS/EA n = 11). One-way ANOVA with a post-hoc Tukey test was used to examine the significance of the mean. ** *p* < 0.01, the RSDS group vs. the control group. # *p* < 0.05, the EA group vs. the RSDS group. ## *p* < 0.01, the EA group vs. the RSDS group. ANOVA: analysis of variance, EA: electroacupuncture, EPM: elevated plus maze, NS: not significant, RSDS: repeated social defeat stress, SEM: standard error of mean

We also used an EPM task to measure anxiety-like behaviors [40]. As shown in Fig. 4A, the trace recorded of RSDS mice exhibited a preference remaining in closed arms compared to open arms during the EPM test. The time spent in the open arms was longer in the RSDS-treated with EA group than in the RSDS group (Fig. 4B; RSDS vs. control, *p* < 0.01; RSDS + sham vs. RSDS, NS; RSDS + EA vs. RSDS, *p* < 0.01; one-way ANOVA). As shown in Fig. 4C, the RSDS group treated with EA reduced the time spent in the closed arms compared to the RSDS group with sham treatment (RSDS vs. control, *p* < 0.01; RSDS + sham vs. RSDS, NS; RSDS + EA vs. RSDS, *p* < 0.05; one-way ANOVA). There was no significant difference in the time spent in the center of the EPM apparatus between the groups (Fig. 4D). In addition, the velocity traveled in the EPM apparatus was not significantly different between the groups (Fig. 4E). Collectively, these results indicate that EA stimulation at the Baihui and Dazhui acupoints prevents RSDS-induced anxiety-like behaviors.

### Effects of EA stimulation at the bilateral Tianzong (SI11) acupoints

To assess the effect of EA stimulation at the Tianzong acupoints in RSDS-treated mice, we performed EA stimulation at the bilateral Tianzong (SI11) acupoints (EA SI11 group). In the social interaction task, the time spent in the interaction zone with the target present was significantly increased in the group treated with RSDS and EA at Baihui (GV 20) and Dazhui (GV 14) acupoints (Additional file 1: Fig. S1A). However, there was no significant difference between the EA stimulation at the bilateral Tianzong (SI11) acupoints and the RSDS group (Additional file 1: Fig. S1A; RSDS vs. control, *p* < 0.001; RSDS + EA vs. RSDS, *p* < 0.01; RSDS + EA SI11 vs. RSDS, NS; one-way ANOVA). In addition, there was no significant difference in the time spent in the interaction zone with the target absent between the groups (Additional file 1: Fig. S1B). Similarly, the group of RSDS-treated with EA at the Baihui (GV 20) and Dazhui (GV 14) acupoints showed a significantly increased social interaction ratio compared with the control group. However, stimulation with EA bilateral Tianzong (SI11) acupoints did not have improved effects in RSDS-treated mice (Additional file 1: Fig. S1C; RSDS vs. control, *p* < 0.01; RSDS + EA vs. RSDS, *p* < 0.01; RSDS + EA SI11 vs. RSDS, NS; one-way ANOVA). In parallel, EA stimulation at the Baihui (GV 20) and Dazhui (GV 14) acupoints improved the time spent in the corner zone with the target present compared to the RSDS group, but this was not observed at the bilateral Tianzong (SI11) acupoints (Additional file 1: Fig. S1D; RSDS vs. control, *p* < 0.01; RSDS + EA vs. RSDS, *p* < 0.01; RSDS + EA SI11 vs. RSDS, NS; one-way ANOVA). There was no significant difference in the time spent in the corner zone with the target absent between the groups (Additional file 1: Fig. S1E).

In the L/D box task, the group of RSDS mice showed less frequency traveling in the light compartment than the control group (Additional file 2: Fig. S2A). Importantly, EA treatment at Baihui (GV 20) and Dazhui (GV 14) increased the frequency of travel spent in the light compartment, but not in EA bilateral Tianzong (SI11) stimulation (Additional file 2: Fig. S2A; RSDS vs. control, *p* < 0.01; RSDS + EA vs. RSDS, *p* < 0.05; RSDS + SI11 vs. RSDS, NS; one-way ANOVA). In addition, RSDS-induced an increased latency to the first entry into the light compartment which was reduced by EA treatment at the Baihui (GV 20) and Dazhui (GV 14) acupoints but not at the bilateral Tianzong (SI11) acupoints. (Additional file 2: Fig. S2B; RSDS vs. control, *p* < 0.001; RSDS + EA vs. RSDS, *p* < 0.05; RSDS + EA SI11 vs. RSDS, NS; one-way ANOVA). Moreover, the decreased cumulative time spent in the light compartment evoked by RSDS was also significantly increased by EA treatment at Baihui (GV 20) and Dazhui (GV 14) but not bilateral Tianzong (SI11) acupoints (Additional file 2: Fig. S2C; RSDS vs. control, *p* < 0.001; RSDS + EA vs. RSDS, *p* < 0.05; RSDS + EA vs. RSDS, NS; one-way ANOVA).

Similarly, EA treatment at the Baihui (GV 20) and Dazhui (GV 14) acupoints increased the time spent in open arms in the EPM task; however, this was not observed in EA stimulation at the bilateral Tianzong (SI11) acupoints (Additional file 3: Fig. S3A; RSDS vs. control, *p* < 0.05; RSDS + EA vs. RSDS, *p* < 0.05; RSDS + SI11 vs. RSDS, NS; one-way ANOVA). In addition, there was no significant difference in the time spent in the center of the EPM task between each group (Additional file 3: Fig. S3B) Collectively, these findings indicate the specific acupoints of preventive effects on EA stimulation at the Baihui (GV 20) and Dazhui (GV 14) acupoints but not in the bilateral Tianzong (SI11) acupoints in RSDS-evoked social avoidance and anxiety-like behaviors.

### EA stimulation at the Baihui (GV 20) and Dazhui (GV 14) acupoints inhibits the astrocyte activation in hippocampus in RSDS-treated mice

**Fig. 5:**
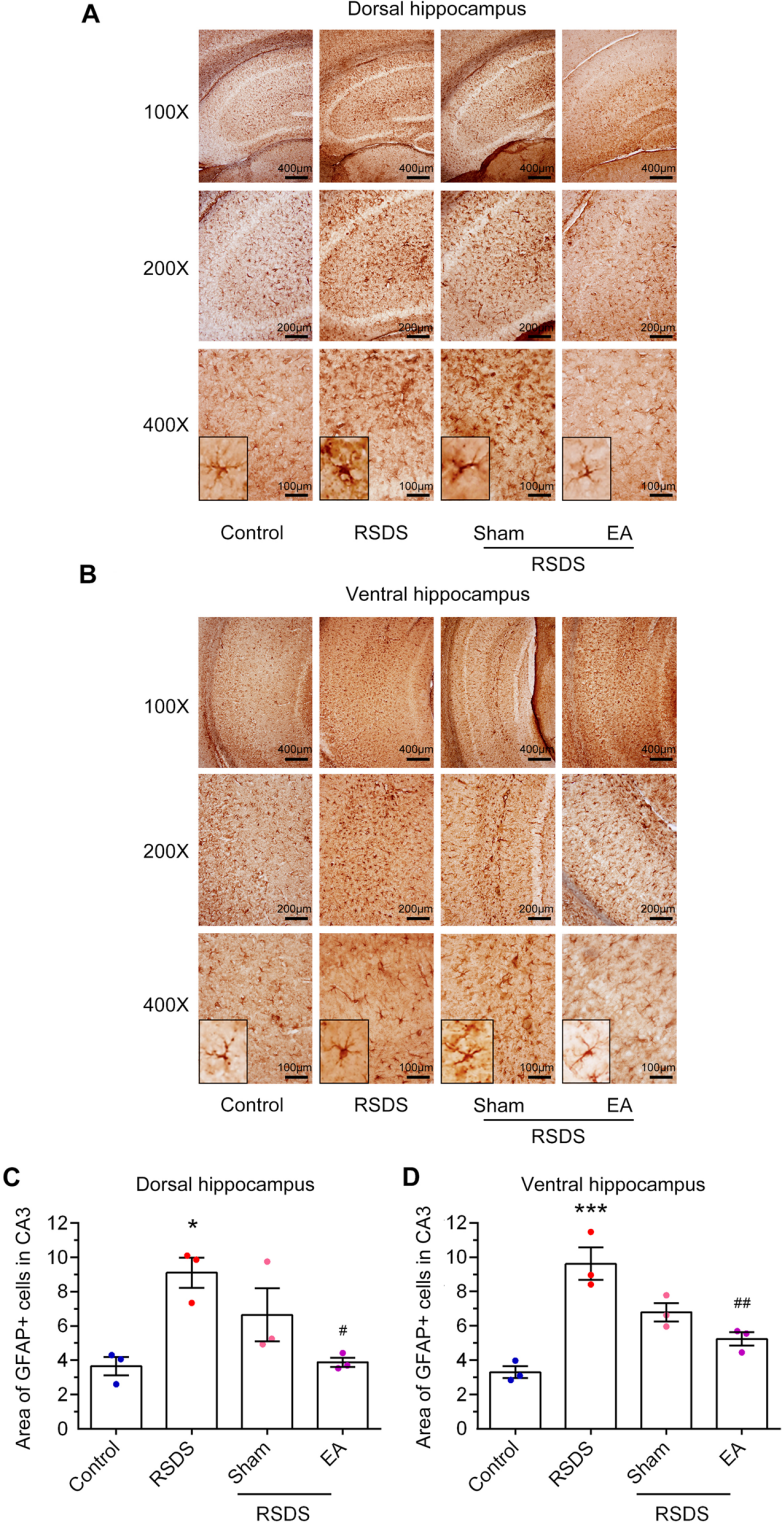
EA stimulation at the Baihui (GV 20) and Dazhui (GV 14) acupoints down-regulates astrocyte activation in RSDS-treated mice. After the behavioral tests, the mice were sacrificed, and brain coronal slices were fixed for immunohistochemistry with an anti-GFAP antibody. Representative images were taken from dorsal (**A**) or ventral (**B**) hippocampal CA3 areas, and GFAP immunoreactivity was quantified as the percentage of GFAP-positive cells from the mice of the control group, RSDS, RSDS with sham treatment, or RSDS with EA treatment. Quantitative results of the dorsal (**C**) and ventral (**D**) hippocampi are presented as the mean ± SEM. (control: n = 16; RSDS: 17; RSDS/Sham: n = 17; RSDS/EA n = 16) One-way ANOVA with a post-hoc Bonferroni test was used to examine the significance of the mean. * *p* < 0.05 vs. the control group. *** *p* < 0.001 vs. the control group. # *p* < 0.05 vs. the RSDS group. ## *p* < 0.01 vs. the RSDS group. ANOVA: analysis of variance, EA: electroacupuncture, GFAP: glial fibrillary acidic protein, RSDS: repeated social defeat stress, SEM: standard error of mean

As shown in Fig. 5A, there were prominent astrogliosis throughout the dorsal hippocampus CA3 region in RSDS-stimulated mice compared with the control groups. In addition, the morphological characteristics of astrocyte activation in the ventral hippocampus were also observed, including a hypertrophic appearance with thick, densely labeled processes, and large cell bodies (Fig. 5B). However, astrocyte activation in the RSDS mice brain were attenuated by EA stimulation, compared to the sham treatment. As shown in Fig. 5C and D, the quantitative results of GFAP immunoreactivity were significantly increased in both the dorsal (RSDS vs. control, *p* < 0.05; one-way ANOVA) and ventral (RSDS vs. control, *p* < 0.001; one-way ANOVA) hippocampus in the RSDS group compared to the control group. Importantly, GFAP immunoreactivity was significantly decreased by EA treatment in the dorsal (Fig. 5C, RSDS + EA vs. RSDS, *p* < 0.05; one-way ANOVA) and ventral (Fig. 5D, RSDS + EA vs. RSDS, *p* < 0.01; one-way ANOVA) hippocampal CA3 region compared to the control group. In addition, the sham treatment had no effect on GFAP immunoreactivity (Fig. 5C and D). Collectively, these data indicate that EA stimulation at the Baihui (GV 20) and Dazhui (GV 14) acupoints prevents RSDS-induced astrocyte activation in the hippocampus.

### EA stimulation at the Baihui (GV 20) and Dazhui (GV 14) acupoints down-regulates lipocalin 2 expression in the hippocampal astrocytes in RSDS-treated mice

**Fig. 6:**
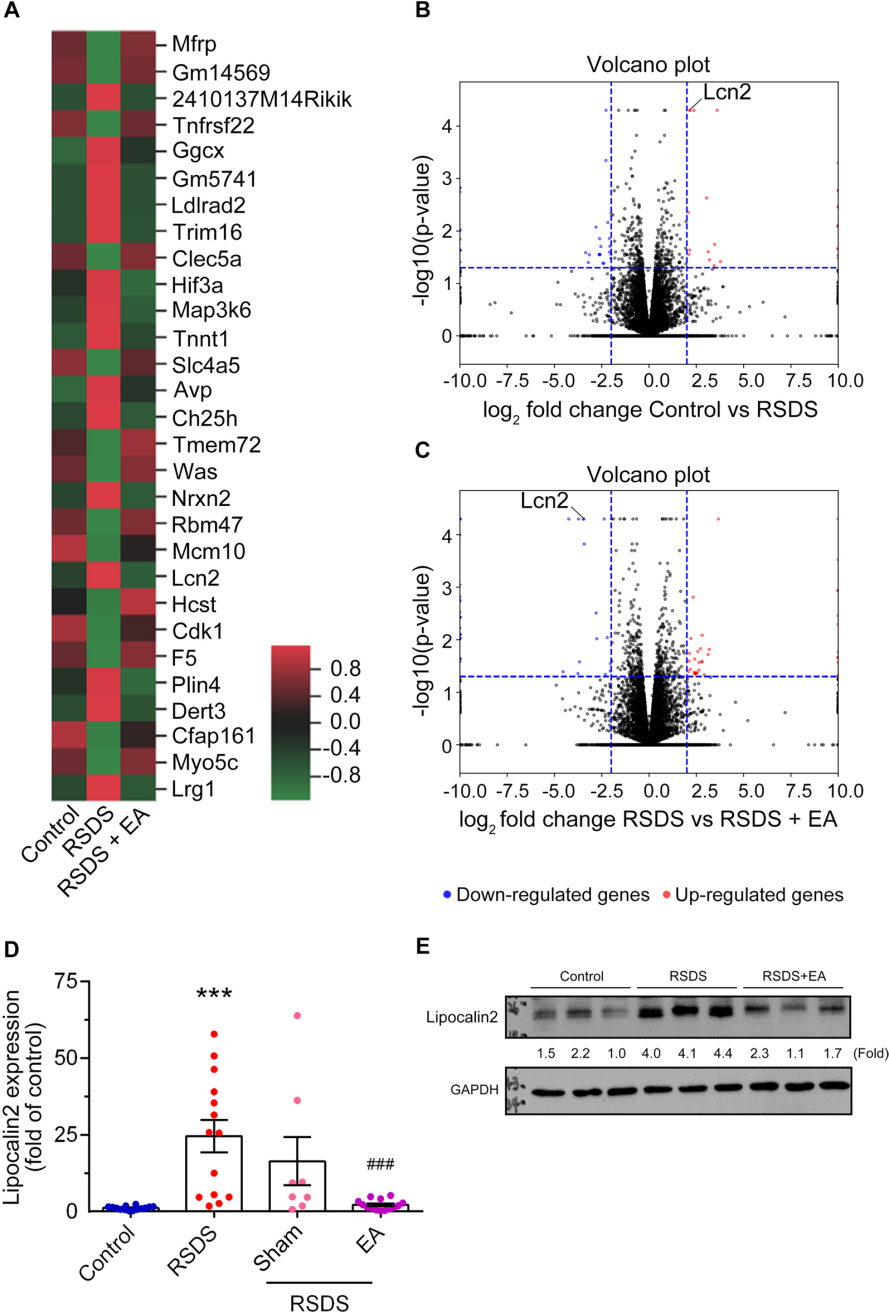
EA stimulation at the Baihui (GV 20) and Dazhui (GV 14) acupoints down-regulates the expression of lipocalin 2 in the hippocampus elicited by RSDS. Hippocampal tissues were collected after the behavior test. **A** Heat map representing the change in gene expression in the control, RSDS, and RSDS-treated with EA groups. Induced gene sets are shown in red, and repressed gene sets are shown in green. Differentially downregulated (blue spots, left panel) and upregulated (red spots, right panel) gene expression profiling with *p* < 0.05, in the control versus RSDS groups (**B**) and RSDS versus RSDS + EA groups (**C**) were identified using the volcano plot. Genes with -log10 (*p*-value) > 1.3, and fold change > 2 were selected for further analyses. mRNA expression (**D**) and protein expression (**E**) of lipocalin 2 were determined by real-time PCR and western blotting, respectively. Quantitative results are presented as the mean ± SEM. One-way ANOVA with a post-hoc Tukey test was used to examine the significance of the mean. *** *p* < 0.001 vs. the control group. ### *p* < 0.001 vs. the RSDS group. ANOVA: analysis of variance, EA: electroacupuncture, PCR: polymerase chain reaction, RSDS: repeated social defeat stress, SEM: standard error of mean

To further identify the mechanism of EA treatment at the Baihui (GV 20) and Dazhui (GV 14) acupoints in RSDS-treated mice, the hippocampal tissue was subjected to genome-wide gene expression profiling using NGS analysis. Gene expression in the mouse hippocampus after EA treatment under RSDS is shown in the heat map representation (Fig. 6A). Gene expression with *p*-value ≤ 0.05, and ≥ twofold changes were considered significantly differentially expressed. Induced gene sets are presented in red, and repressed gene sets are presented in green (Fig. 6A). A volcano plot of gene expression with *p* < 0.05 (fold change in relative expression as determined by log2, with upregulated genes are shown in red spots and downregulated genes in blue spots) in the control group versus the RSDS mice (Fig. 6B), and RSDS mice versus RSDS mice treated with EA stimulation (Fig. 6C). Analysis of the differentially expressed genes (DEGs) regulated by RSDS pointed out *Ggcx, Gm10222, Rnf181* and *Lipocalin 2 (Lcn2)* were significantly upregulated and three gene (*F5, Rps27,* and *Srsf1*) were downregulated, compared with control group (Additional file 4: Fig. S4A). However, there were five genes (*Gm11637, Usp39, Rbs6kb2, Tmem72,* and *F5*) were significantly upregulated, and eighteen genes were downregulated by EA treatment compared with the RSDS group (Additional file 4: Fig. S4B). Remarkably, among the eighteen genes only two genes (*Gm10222* and *Lcn2*) were significantly upregulated by RSDS, but downregulated by EA treatment (Additional file 4: Fig. S4A and B). Particularly, protein-interaction network analysis, as analyzed by the size and depth of each node which is positively correlated to the degree of protein connection to others, identified the *Lcn2* as the major relevant signaling molecule (Additional file 5: Fig. S5). We focused on one inducible molecule: lipocalin 2, which secreted by reactive astrocytes was upregulated in the hippocampus following psychological stress [41]. We also found the distribution of *Lcn2* transcripts using a volcano plot in which *Lcn2* was upregulated in the RSDS group compared with the control group (Fig. 6B). In contrast, expression of the *Lcn2* gene was reduced following EA treatment in the RSDS group (Fig. 6C). As presented in Additional file 6: Fig. S6, the top five pathways enriched from KEGG database were found the subset genes S100A8 (calgranulin A or migration inhibitory factor-related protein 8; MRP-8) and S100A9 (calgranulin B, or MRP-14) of *Lcn2* and which relevant pathway: interleukin (IL)-17 signaling pathway (Additional file 6: Fig. S6). Interestingly, modulating transcriptome including *S100a8* and *S100a9* genes have been observed that associated with behavioral response to chronic mild stress stimulus [42]. In addition, the hippocampal transcriptome of mice subjected to chronic social stress also pointed out that S100a8 and S100a9 genes affected inflammation were upregulated in the hippocampus [43]. Importantly, the immune-response genes of *Lcn2, S100a8,* and *S100a9* have been found that markedly increased in the patients’ hippocampus with Alzheimer's disease [44]. Recently, progressive multiple sclerosis model caused depression- and anxiety-like behaviors, which has been found simultaneously upregulation of IL-17 and GFAP in the hippocampus but not prefrontal cortex [45]. The recent study also reported that cumulative mild stress promoted depression-like behaviors in young adult mice and specifically upregulated IL-17 expression in the hippocampus [46]. We then further verified whether lipocalin 2 expression in RSDS was reduced by EA stimulation. Our results showed that RSDS-induced the upregulation of lipocalin 2 mRNA (Fig. 6D; RSDS vs. control, *p* < 0.001; one-way ANOVA) and protein expression (Fig. 6E) in the hippocampus of RSDS mice compared to those in the control group. Conversely, the enhancement of lipocalin 2 was dramatically reduced in the EA group compared to that in the RSDS mice (Fig. 6D; RSDS + EA vs. RSDS, *p* < 0.001; one-way ANOVA). In addition, lipocalin 2 levels in the hippocampus were not significantly different between the RSDS and sham treatment groups (Fig. 6D, RSDS + sham vs. RSDS, NS; one-way ANOVA).

**Fig. 7:**
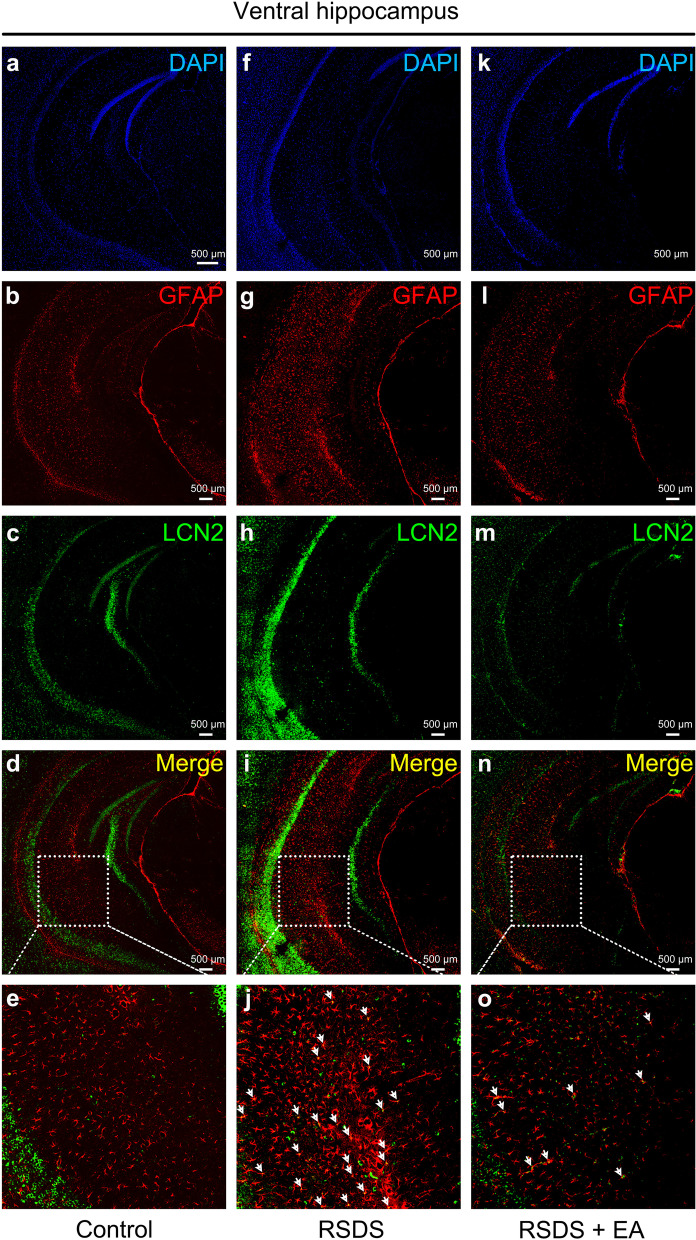
EA stimulation at the Baihui (GV 20) and Dazhui (GV 14) acupoints down-regulates the expression of lipocalin 2 in reactive astrocytes in RSDS-treated mice. Double-labeling immunofluorescence of lipocalin 2 (green) and GFAP (red) in the control (**a**–**e**), RSDS (**f**–**j**) and RSDS-treated mice with EA stimulation (**k**–**o**) in the ventral hippocampus of the brain sections. DAPI staining (blue) was used to label the nuclei. The merged yellow images indicate co-localization of lipocalin 2 and GFAP (white arrowheads). (3 animals in each group) All images were taken at a primary magnification of 200 × , and the high magnification (1000×) of **d**, **i** and **n** are shown in **e**, **j**, and **o**, respectively. EA: electroacupuncture, RSDS: repeated social defeat stress, GFAP: glial fibrillary acidic protein, LCN2: lipocalin 2

Next, we assessed the distribution of lipocalin 2 expression by double labeling for immunofluorescence, counterstained with DAPI (Fig. 7a, f, k), and analyzed by confocal microscopy. There was a low expression of GFAP (Fig. 7b) and lipocalin 2 (Fig. 7c–e) staining in the ventral hippocampus of the mice in the control group. Importantly, both the increase in GFAP (Fig. 7g) and lipocalin 2 (Fig. 7h and i) staining were observed in the RSDS group. Moreover, the increase lipocalin 2 was co-localized in GFAP-positive astrocytes (approximately 3.6-fold) in the ventral hippocampus in the RSDS group (Fig. 7i and j). In contrast, EA stimulation decreased GFAP (Fig. 7l) and lipocalin 2 (Fig. 7m) staining in the ventral hippocampus. Importantly, the co-localization of lipocalin 2 and GFAP was also reduced to approximately 19.7% by EA stimulation (Fig. 7n and o). Collectively, our results suggest that lipocalin 2 may be a key regulatory factor for EA treatment in RSDS mice.

### Overexpression of lipocalin 2 protein in the brain causes social avoidance and anxiety-like behaviors

**Fig. 8:**
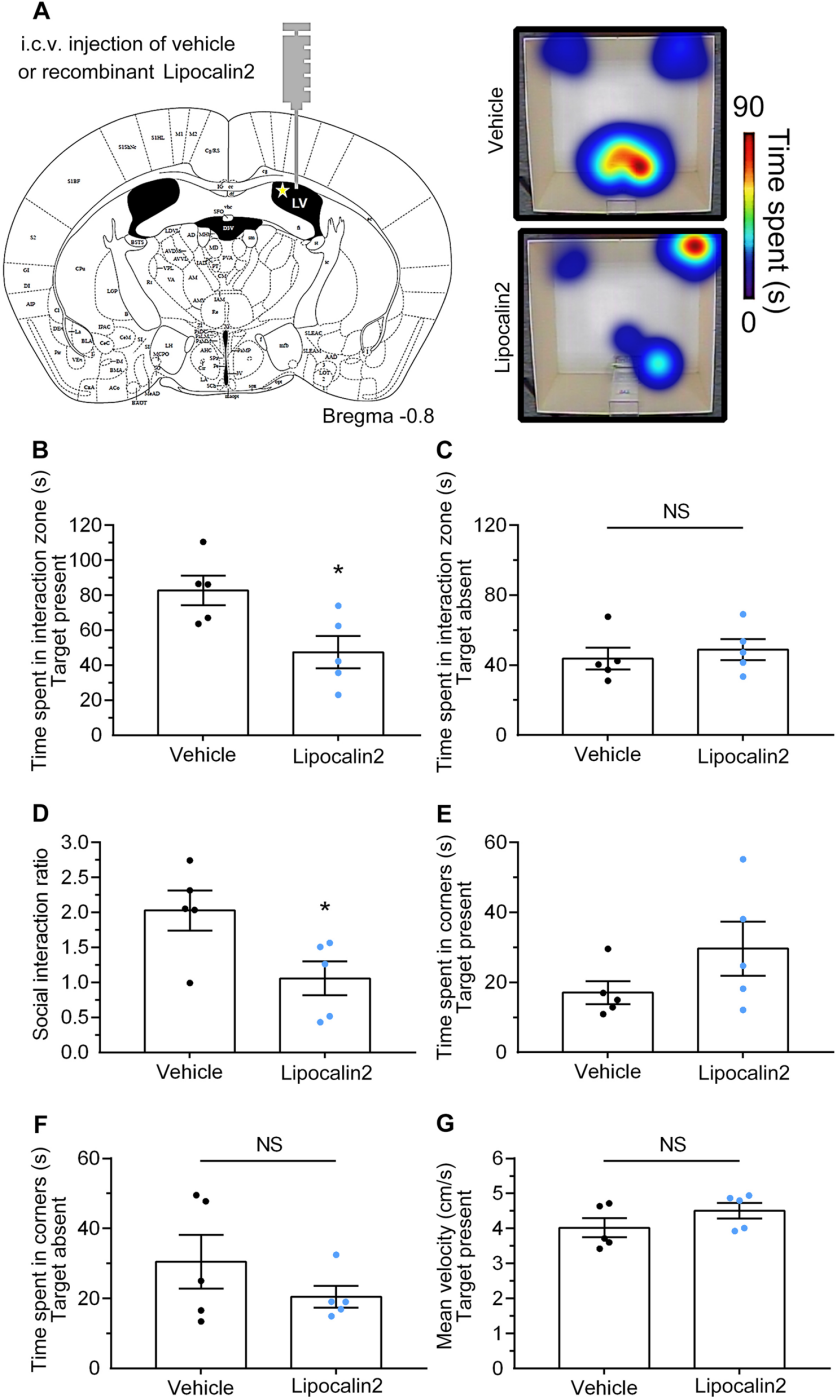
Overexpression of lipocalin 2 in the brain induces social avoidance**.** Single i.c.v. injection of recombinant lipocalin 2 protein (7 μL and 1 μg/mL) or vehicle in the brain of the mice and social interaction behavioral tests were performed after 6 days. **A** i.c.v. injection position in lateral ventricles (left panel), and the representative heat maps were shown in right panel. The time spent in the interaction zone while the target was present (**B**) or absent (**C**). **D** The social interaction ratio (the time spent in the interaction zone while the target was absent / the time spent in the interaction zone while the target was present). The time spent in corners while the interaction zone with (**E**) or without (**F**) the target mice. **G** The velocity traveled in the target apparatus. Quantitative data are presented as the mean ± SEM (n = 5 each group). The Student’s t-test was used to examine the significance of the mean. * *p* < 0.05 vs. the vehicle or control groups. i.c.v.: intracerebroventricular, LCN2: lipocalin 2, NS: not significant, SEM: standard error of mean

**Fig. 9:**
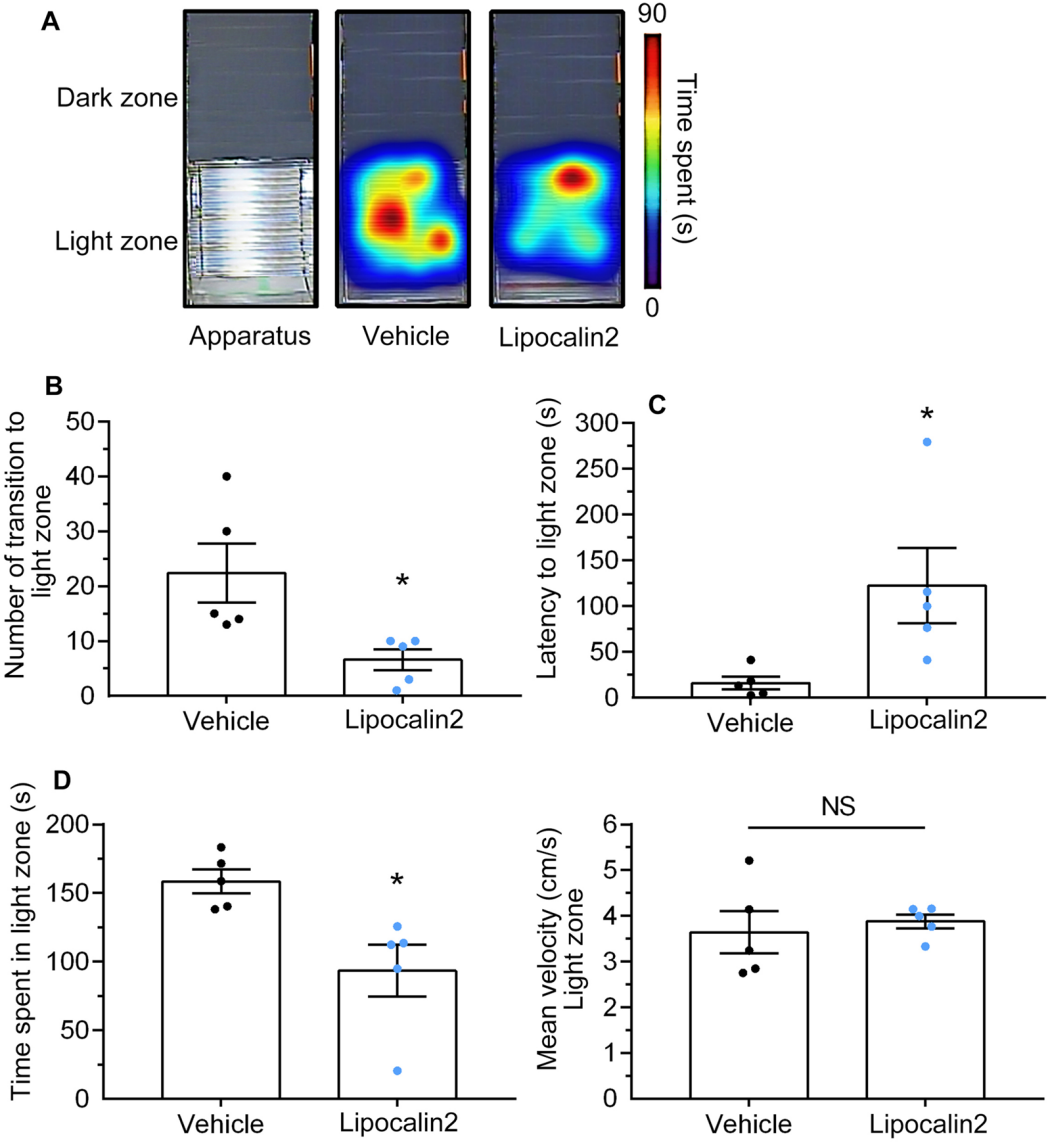
Overexpression of lipocalin 2 in the brain induces anxiety-like behaviors in the light and dark task. Single intracerebroventricular injection of recombinant lipocalin 2 protein (7 μL, 1 μg/mL) or vehicle in the brain of the mouse, and light and dark tasks were assessed after 6 days. **A** Representative heat maps for the activities of each group during the light–dark test. The frequency during the time spent in the light compartment (**B**), the latency to the first entry into the light compartment (**C**), or the cumulative time in the light compartment (**D**) were determined. The velocity traveled in the light compartment is measured in (**E**). Quantitative data are presented as mean ± SEM (n = 5 per group). The Student’s t-test was used to examine the significance of the mean. * *p* < 0.01, vs. the vehicle or control group. LCN2: lipocalin 2, NS: not significant, SEM: standard error of mean

**Fig. 10:**
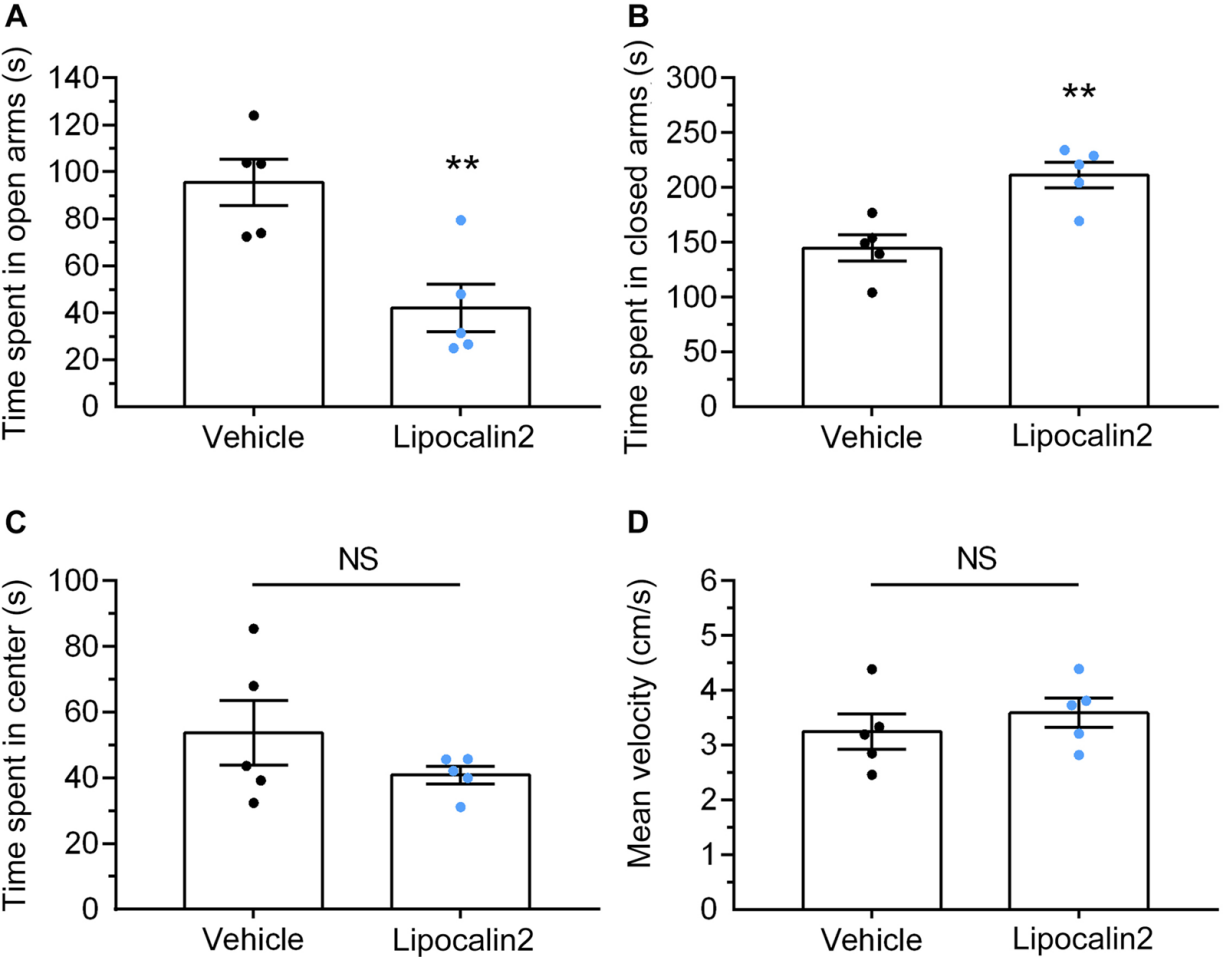
Overexpression of lipocalin 2 in the brain induces anxiety-like behaviors in the EPM task. Single intracerebroventricular injection of recombinant lipocalin 2 protein (7 μL, 1 μg/mL) or vehicle in the brain of the mice and the EPM task were performed after 6 days. Time spent in the open arms (**A**), closed arms (**B**), or center (**C**) of the EPM apparatus. **D** Velocity traveled in the EPM apparatus. Quantitative data are presented as mean ± SEM (n = 5 per group). The Student’s t-test was used to examine the significance of the mean. ** *p* < 0.01 vs. the vehicle group. EPM: elevated plus maze, LCN2: lipocalin 2, NS: not significant, SEM: standard error of mean

To determine whether increasing lipocalin 2 in the brain associates RSDS-induced negative behaviors, C57/BL6 mice received an i.c.v. injection of mouse recombinant lipocalin 2 protein on a stereotaxic apparatus (Fig. 8A, left panel), and behavioral tasks were measured after 6 days. The right panel of Fig. 8A shows the representative heat maps of the social interaction analysis following RSDS. In the social interaction test, the time spent in the social interaction zone (Fig. 8B) and the social interaction ratio (Fig. 8D) were significantly reduced in lipocalin 2-injected mice compared to vehicle-injected mice. Conversely, the lipocalin 2 injected mice had a tendency to increase the time spent in the corner (Fig. 8E). In addition, there was no significant difference in the time spent in the social interaction zone (Fig. 8C) or corners (Fig. 8F), with the target mice being absent. The velocity traveled by both the lipocalin 2- and vehicle-injected mice was also not significantly different (Fig. 8G). Figure 9A shows the representative heat maps of the light compartment in the L/D box task. The lipocalin 2-injected mice had a low frequency when traveling in the light compartment, compared with the vehicle-injected mice (Fig. 9B; lipocalin 2 vs. vehicle, *p* < 0.05; Student’s t-test). Surprisingly, lipocalin 2-injected mice had a longer latency to the first entry into the light compartment (Fig. 9C; lipocalin 2 vs. vehicle, *p* < 0.05; Student’s t-test). Moreover, the cumulative time spent in the light compartment was decreased in the lipocalin 2-injected mice compared to the vehicle group (Fig. 9D; lipocalin 2 vs. vehicle, *p* < 0.05; Student’s t-test). In addition, the velocities traveled by these two groups were not significantly different (Fig. 9E). We also performed the EPM task to further determine cognitive impairment in lipocalin 2 injected mice. As shown in Fig. 10A, lipocalin 2-treated mice spent less time in the open arms than the vehicle group (lipocalin 2 vs. vehicle, *p* < 0.01; Student’s t-test). In parallel, the lipocalin 2-injected mice spent more time in the closed arms than the vehicle-injected mice (Fig. 10B, lipocalin 2 vs. vehicle, *p* < 0.01; Student’s t-test). In addition, there were no significant differences observed in the time spent in the center (Fig. 10C) and the velocity traveled (Fig. 10D) in the EPM apparatus in both groups. Collectively, our results suggest that lipocalin 2 overexpression in the brain may provoke cognitive impairment, including social avoidance and anxiety-like behaviors, similar to RSDS-treated mice.

### Overexpression of lipocalin 2 protein in the brain alters astrocyte activation and stress-related gene expression

**Fig. 11:**
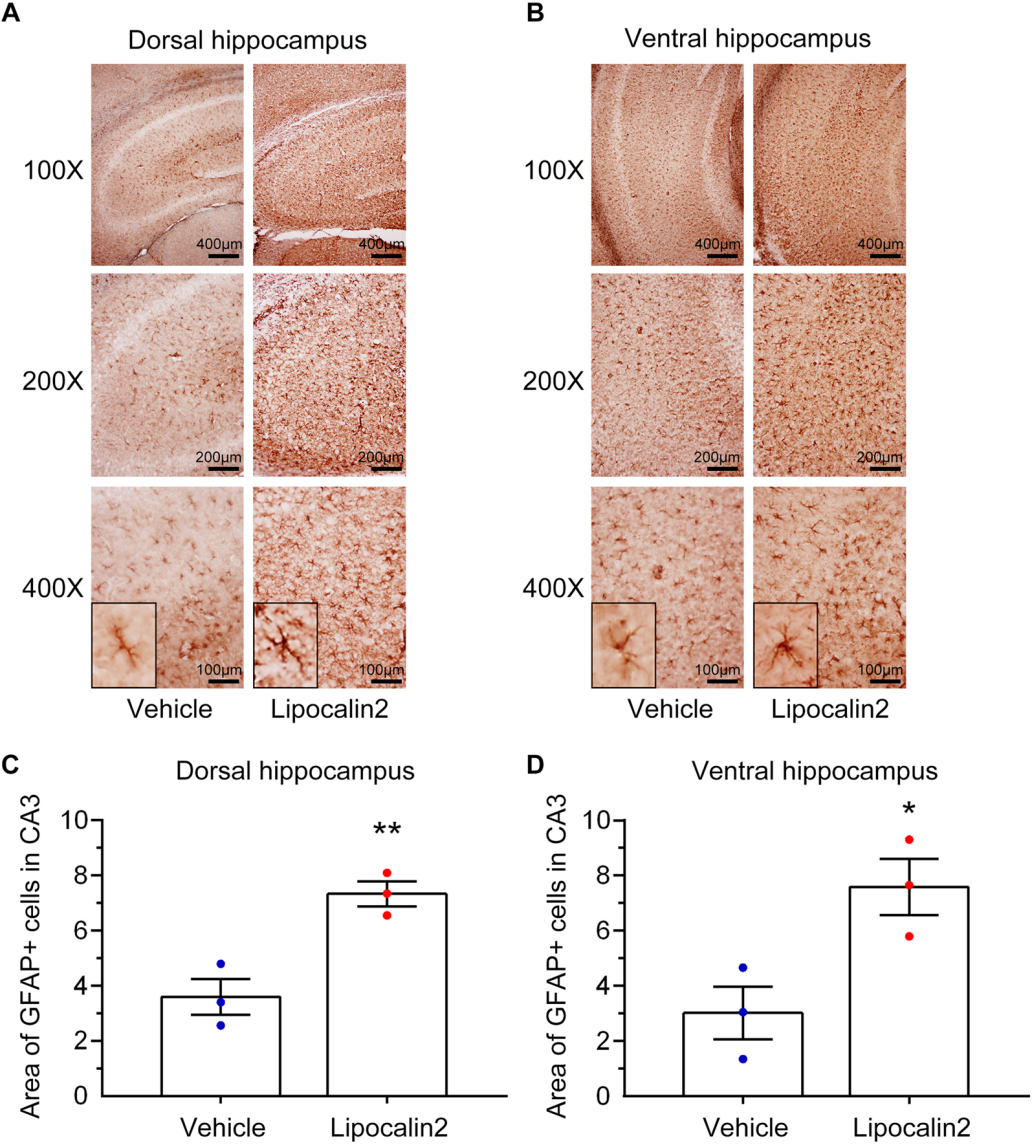
Overexpression of lipocalin 2 in the brain induces astrocyte activation in the hippocampus. After a single intracerebroventricular injection of recombinant lipocalin 2 protein (7 μL, 1 μg/mL) or vehicle in the brain of the mice for 7 days, the brain coronal slices were fixed for immunohistochemistry with anti-GFAP antibody. Representative images were taken from the dorsal (**A**) and ventral (**B**) hippocampal CA3 areas, and the GFAP immunoreactivity was quantified as the percentage of GFAP-positive cells in the dorsal (**C**) and ventral (**D**) hippocampi of the vehicle and lipocalin 2 groups. Quantitative results are presented as mean  ±  SEM (n =  3 per group). The Student’s t-test was used to examine the significance of the mean. * *p* <  0.05 vs. the vehicle group. ** *p*  <  0.01 vs. the vehicle group. GFAP: glial fibrillary acidic protein, LCN2: lipocalin 2, SEM: standard error of mean

**Fig. 12:**
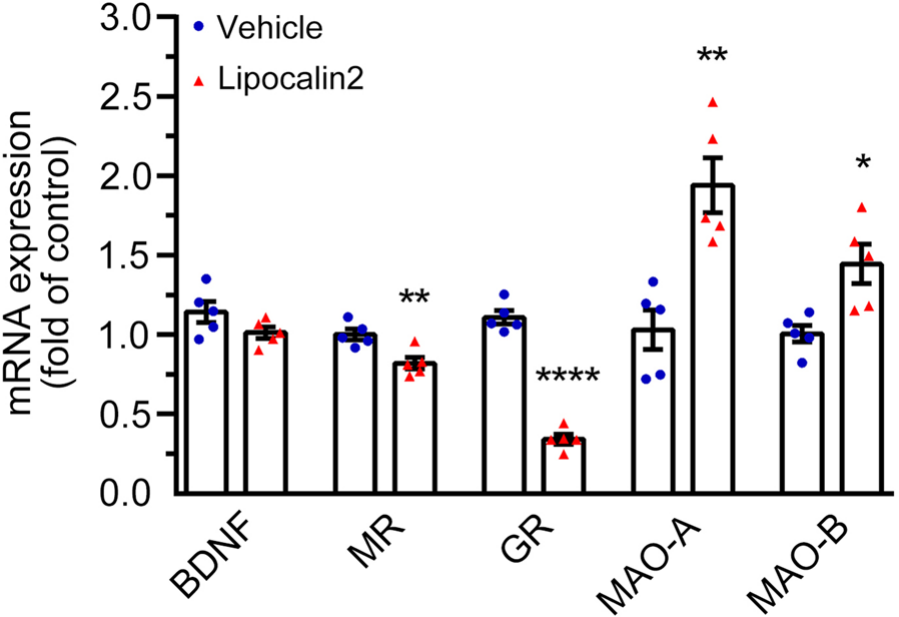
Overexpression of lipocalin 2 in the brain alters stress-related gene expression in the hippocampus. After a single intracerebroventricular injection of recombinant lipocalin 2 protein (7 μL, 1 μg/mL) or vehicle in the brain of the mice for 7 days, the hippocampal tissues were collected for mRNA assay. The expression of MR, GR, MAO-A, or MAO-B was determined by real-time PCR. Quantitative data are presented as mean ± SEM (n = 5 per group). The Student’s t-test was used to examine the significance of the mean. * *p* < 0.05 vs. the vehicle group. ** *p* < 0.01 vs. the vehicle group. **** *p* < 0.001 vs. the vehicle group. GR: glucocorticoid receptor, MAO-A: monoamine oxidase A, MAO-B: monoamine oxidase B, MR: mineralocorticoid receptor, PCR: polymerase chain reaction, SEM: standard error of mean

We further assessed whether increasing lipocalin 2 in brain alters the activation of astrocytes and stress-related genes. Astrocytes in the lipocalin 2 group showed morphological characteristics of activation in the dorsal (Fig. 11A) or ventral (Fig. 11B) hippocampus after the mice received i.c.v. injection of mouse recombinant lipocalin 2 protein. The quantitative results showed that the GFAP immunoreactivity was significantly increased in both the dorsal (Fig. 11C, lipocalin 2 vs. vehicle, *p* < 0.01; Student’s t-test) and ventral (Fig. 11D, lipocalin 2 vs. vehicle, *p* < 0.05; Student’s t-test) hippocampal CA3 region in the lipocalin 2-injected mice as compared to the vehicle group. Furthermore, we examined whether increasing lipocalin 2 alters stress-related gene expression in the hippocampus. As shown in Fig. 12, the expression of MR (lipocalin 2 vs. vehicle, *p* < 0.01; Student’s t-test) or GR (lipocalin 2 vs. vehicle, *p* < 0.0001; Student’s t-test) was significantly lower in the lipocalin 2 group than in the vehicle group. On the other hand, the expression of MAO-A (lipocalin 2 vs. vehicle, *p* < 0.01; Student’s t-test) or MAO-B (lipocalin 2 vs. vehicle, *p* < 0.05; Student’s t-test) was dramatically increased in the lipocalin 2 group compared to the vehicle group. Collectively, these results indicate that increasing lipocalin 2 in the brain promotes astrocyte activation and alters stress-related gene expression in the hippocampus, which may cause behavioral impairment.

## Discussion

Acupoint specificity means acupuncture at acupoints induces a more efficient therapeutic response than non-acupoint. Particularly, there are three properties of special structures beneath acupoints have been supported including muscle-spindle-rich area, cutaneous-receptor rich area, and tendon-organ-rich area [47]. The proportional locations of the acupoints in mouse in this study were determined following the anatomical description in the WHO guidelines for human acupoints [48, 49]. There are only few studies have reported the effects of EA treatment in an animal model of sociability. Laser acupuncture at Shenmen (HT7) in a postnatal autistic mouse model improved autistic-like behaviors, including learning, memory, and social behavior [50]. Moreover, stimulation at the catgut acupoint in a social isolation mouse model is effective in reversing depression-like behavior [51]. EA treatment at the Zusanli (ST36) acupoint has also been reported to reverse the memory impairments of isolated mice [52], and to improve the social behaviors in the low socially interacting rats [53]. EA has previously reported to improve PTSD-like behaviors in rats by enhancing hippocampal endocannabinoid signaling [54]. Recently, several preclinical evidence indicated that Baihui (GV20) acupoint is most frequently used in PTSD [27]. In Chinese medicine theory, these acupoints are on the “Du meridian”, involves direct communication with the brain. Moreover, the Dazhui (GV14) and Baihui (GV20) commonly used acupoints in traditional Chinese medicine to treat neurological disorders. The stimulation of EA at the Dazhui (GV14) and Baihui (GV20) acupoints exerts neuroprotective effects in cerebral ischemia–reperfusion injury in rats [55, 56]. In a rodent model, EA treatment at the Dazhui (GV14) and Baihui (GV20) acupoints also exerts neuroprotective effects in cerebral ischemia–reperfusion injuries [55, 56]. In the clinic, acupuncture treatment at the Dazhui (GV14) and Baihui (GV20) acupoints effectively improved the symptoms of infantile primary and Jacksonian epilepsy [57, 58]. In the present study, EA treatment at the Dazhui (GV14) and Baihui (GV20) acupoints reduced RSDS-induced anxiety-like behaviors and social avoidance, which possess potent therapeutic effects in mood disorders. Importantly, our results also support previous studies that stimulation of bilateral Tianzong (SI11) acupoints could be sham acupoints in an acupuncture study.

Lipocalin 2 (also known as neutrophil gelatinase-associated lipocalin [NGAL]) in the CNS is predominantly secreted by astrocytes under inflammatory conditions and promotes morphological transformation and migration in astrocytes [59]. Moreover, lipocalin 2 has been reported to be released by injured neurons as a “help-me” distress signal that activates the astrocytes into potentially pro-recovery phenotypes [60]. Recently, lipocalin 2 has been shown to play an important role in pathological conditions, specifically its association with cognition during the progression of pathology [61]. Importantly, lipocalin 2 has previously been found to be upregulated in the hippocampus following psychological stress and regulates stress-induced neuronal excitability and anxious behavior [62], which has been considered as a potential biomarker in aging-related cognitive decline [63]. Lipocalin 2-null mice are reported to display anxious and depressive-like behaviors, as well as cognitive and memory impairment [64]. A recent report also suggests that lipocalin 2 secreted from reactive astrocytes in the cerebrospinal fluid is a promising biochemical marker candidate for the differential diagnosis of vascular dementia and neurodegenerative dementias [65]. A recent study also reported that astrocytes from patients with Parkinson’s disease are more responsive to lipocalin 2 stimulation for reactive astrocytosis [66]. Higher lipocalin 2 expression in patients brain has been found that significantly associated with depression scores [67]. Moreover, elevated plasma lipocalin 2 levels is also been reported that associated in depressed older patients with a pathological waist circumference [68]. Interestingly, lipocalin 2 upregulation has also been reported that associated with sex-specific cognitive functioning in late-life depression [69]. Our results revealed that astrocyte activation and lipocalin 2 expression induced by RSDS were effectively reduced by EA treatment at the Baihui (GV 20) and Dazhui (GV 14) acupoints. Importantly, overexpression of lipocalin 2 in the brain also elicited PTSD-like behaviors, such as social avoidance and anxiety-like behaviors. Our findings support the previous reports that the expression of lipocalin 2 in the brain may be an important regulator of cognitive symptoms, and increased lipocalin 2 levels is associated with depression or PTSD.

The hippocampus is particularly vulnerable to chronic stress, if impaired, leading to mood disorders such as anxiety-like and depressive-like behaviors [70]. There are at least two distinct functional regions of the hippocampus, namely the ventral and dorsal hippocampi. The dorsal region serves for memory and learning of conceptual information, but the ventral hippocampus primarily modulates emotional processes, such as fear and anxiety [71]. Particularly, ventral hippocampus has also been reported which is a specific region regulates susceptibility to chronic social defeat stress [72], and may act as a potential key mediator of stress-induced anxiety-like behavior [73]. Surprisingly, the lipocalin 2 null mice have been found that the ventral hippocampus were hypertrophic and display anxious and depressive-like behaviors [64]. The dorsal hippocampal CA1 neural systems have also been reported to modulate anxiety behaviors [74]. Remarkably, the hippocampal CA3 region is a crucial role in the stress response that exerts a negative feedback in the regulation of the HPA axis [75]. In addition, the hippocampal CA3 is also a region of dynamic stress-induced structural plasticity [76]. Chronic stress has been proposed to induce hippocampal CA3 neuronal retraction, which then impairs activity of the HPA axis, leading to disturbed glucocorticoid release [77]. Particularly, chronic glucocorticoid treated animals have also been observed that remodeling the CA3 pyramidal neurons but not the CA1 pyramidal cells or the granule cells of the dentate gyrus [78]. Moreover, CA3 region has been observed that is more vulnerable and experts earlier response of adhesion properties that cerebral cortex and CA1 region under ischemia challenge [79]. It has also been reported that stress-induced dendritic retraction of CA3 pyramidal neurons has been implicated as mechanisms contributing to hippocampal shrinkage [80].

Decreased levels of endogenous anti-inflammatory proteins Nrf2 and Keap1 in the CA3 hippocampus of mice have been implicated in the pathophysiology of depression after social defeat stress [81]. Moreover, infusion of BDNF recombinant protein or a TrkB agonist into the CA3 hippocampus promoted long-lasting antidepressant effects in mice models of depression [82]. Otherwise, Nrf2 knockout mice have been found that reveal lower BDNF and its receptor TrkB signaling and higher inflammation in the CA3, which also show depression-like phenotypes than those of WT mice [83]. Single prolonged stress-enhanced astrocyte activation in the rat brain could last a few days, which increasing the vulnerability of abnormal fear and learning [84]. GFAP expression in brain may suggest a response of exposure to stress [85]. Remarkably, GFAP has been reported that plays a crucial role in CA3 neuronal survival after injury [86]. Importantly, the populations of astrocytes have been observed that increased in the CA3 hippocampus after spatial learning in rats [87]. Furthermore, the neonatal mice subjected to maternal separation presented an increased activation of astrocytes in the CA3 area [88]. The activity-stress rats have been observed increased significantly GFAP-immunoreactive cells in the hippocampal CA3 region, which is similar to the response found in ischemia [89]. Therefore, this study has focused on a specific marker expression in the vulnerable brain region responds to social-defeat stress challenge to access whether EA treatment improves RSDS-responsive expression of GFAP and astrocyte-associated lipocalin 2 in the hippocampal CA3 region.

As a general rule, a malfunction of the HPA axis has been strongly implicated in the development of mood disorders under stress [90]. There are two types of adrenal steroid receptors in the hippocampus: MR and GR. MR plays an important role in negative feedback and affects hippocampal function in patients with PTSD [91]. Furthermore, downregulation or disruption of GR expression in the hippocampus has been found to enhance depression and anxiety behaviors [92]. Our previous results showed that the injection of a GR antagonist impaired social behavior [11]. On the other hand, treatment options for depressive and anxious patients are based on the monoamine hypothesis and aim to increase the availability of monoamines [93]. Both MAO-A and MAO-B modulate neurobiochemistry by degrading monoamine neurotransmitters, including serotonin, dopamine, and norepinephrine [94]. A significant increase in the monoamine oxidase MAO-A is found in association with the pathogenesis of major depressive disorders [95], and selective serotonin reuptake inhibitor treatment elevated MAO-A binding in the brain regions of patients with depression, which contributes to recurrence [96]. In previous studies, C57BL/6 mice were subjected to the RSDS model to induce social avoidance and chronic anxiety-like behaviors, and alter the expression of stress-related genes in the brain [97]. Our previous study also reported that chronic social defeat stress induces social avoidance behavior and increases MAO-A and MAO-B expression in mice [11]. The present study found that overexpression of lipocalin 2 in the brain evoked PTSD-like behaviors such as social avoidance and anxiety-like behaviors. These results also found that overexpression of lipocalin 2 in the brain induced astrocyte activation, decreased MR and GR, and increased the expression of MAO-A and MAO-B.

## Conclusions

We established a preclinical repeated social defeat mouse model, which develops symptoms of PTSD similar to humans, such as social avoidance, despair, and anxiety. The present study suggests that the stimulation of EA, but not needle insertion at the Baihui (GV 20) and Dazhui (GV 14) acupoints effectively improves RSDS-elicited social avoidance and anxiety-like behaviors. Moreover, astrocyte activation and lipocalin 2 expression induced by RSDS were effectively reduced by EA treatment at the Baihui (GV 20) and Dazhui (GV 14) acupoints. Furthermore, overexpression of lipocalin 2 in the brain also elicited PTSD-like behaviors, such as social avoidance and anxiety-like behaviors. Our findings suggest that EA stimulation at the Baihui (GV 20) and Dazhui (GV 14) acupoints effectively improves RSDS-elicited social avoidance and anxiety-like behaviors, and upregulation of lipocalin 2 in the brain may be an important biomarker for the development of PTSD-related symptoms.

## Supplementary Information

**Additional file 1: Fig. S1.** Effects of electroacupuncture stimulation at the bilateral Tianzong (SI11) acupoints in the social interaction task. After RSDS, the experimental mice were introduced into the social interaction test apparatus. The time spent in the interaction zone was determined while the target was present (A) or absent (B). (C) The social interaction ratio was calculated by dividing the time spent in the interaction zone with the target present by the time spent in the interaction zone with the target absent. The time spent in the corners was determined by the interaction zone with the target (D) or without the target (E). Quantitative data are presented as the mean ± SEM (n = 5 each group). One-way ANOVA with a post-hoc Tukey’s test was used to examine the significance of the mean. ** *p* < 0.01 vs. the control group. *** *p* < 0.001 vs. the control group. ## *p* < 0.01 vs. the RSDS group. ANOVA: analysis of variance, EA: electroacupuncture, NS: not significant, RSDS, repeated social defeat stress, SEM, standard error of mean.

**Additional file 2: Fig. S2.** Effects of EA stimulation at the bilateral Tianzong (SI11) acupoints in the light–dark task. After RSDS, the experimental mice were placed in a light–dark task apparatus. The frequency spent in the light compartment (A), the latency to the first light compartment (B), or the cumulative time in the light compartment (C) were determined. Quantitative data are presented as the mean ± SEM (n = 5 each group). One-way ANOVA with a post-hoc Tukey’s test was used to examine the significance of the mean. ** *p* < 0.01 vs. the control group. *** *p* < 0.001 vs. the control group. # *p* < 0.05 vs. the RSDS group. ANOVA: analysis of variance, EA: electroacupuncture, RSDS, repeated social defeat stress, SEM, standard error of mean

**Additional file 3: Fig. S3.** Effects of EA stimulation at the bilateral Tianzong (SI11) acupoints in the EPM task. After RSDS, the experimental mice were introduced into the EPM apparatus. The time spent in the open arms (A) and center (B) of the apparatus were determined. Quantitative data are presented as the mean ± SEM (n = 5 each group). One-way ANOVA with a post-hoc Tukey’s test was used to examine the significance of the mean. * *p* < 0.05, RSDS group compared to the control group. # *p* < 0.05, EA group compared to the RSDS group. ANOVA: analysis of variance, EPM: elevated plus maze, EA: electroacupuncture, NS: not significant; RSDS, repeated social defeat stress.

**Additional file 4:** The bar chart of DEG expression. (A) The log2 ratio of DEG expression in RSDS VS Control. (B) The log2 ratio of DEG expression in EA treatment VS RSDS.

**Additional file 5:** Protein–protein interaction network generated from GeneMANIA analysis. The size of each node presents the degree of connectivity in PPI network, and large node shares more connection to other nodes.

**Additional file 6:** Top five pathways prediction of DEGs by KEGG.

## Data Availability

The data that support the findings are not publicly available. Data are available from the authors upon reasonable request.

## Abbreviations

PTSD: Post-traumatic stress disorder
EA: Electroacupuncture
CNS: Central nervous system
RSDS: Repeated social defeat stress
IL: Interleukin
TNF: Tumor necrosis factor
GFP: Green fluorescent protein
EPM: Elevated plus maze
L/D: Light and dark
GFAP: Glial fibrillary acidic protein
PCR: Polymerase chain reaction
MR: Mineralocorticoid receptor
GR: Glucocorticoid receptor
Ct: Cycle threshold
ANOVA: Analysis of variance
HPA: Hypothalamus–pituitary–adrenal
