# Supplementary figures and images for: Electroacupuncture improves repeated social defeat stress-elicited social avoidance and anxiety-like behaviors by reducing Lipocalin-2 in the hippocampus
Source: Mol Brain. 2021 Sep 26;14:150. doi: 10.1186/s13041-021-00860-0 (PMC8474847; doi:10.1186/s13041-021-00860-0)

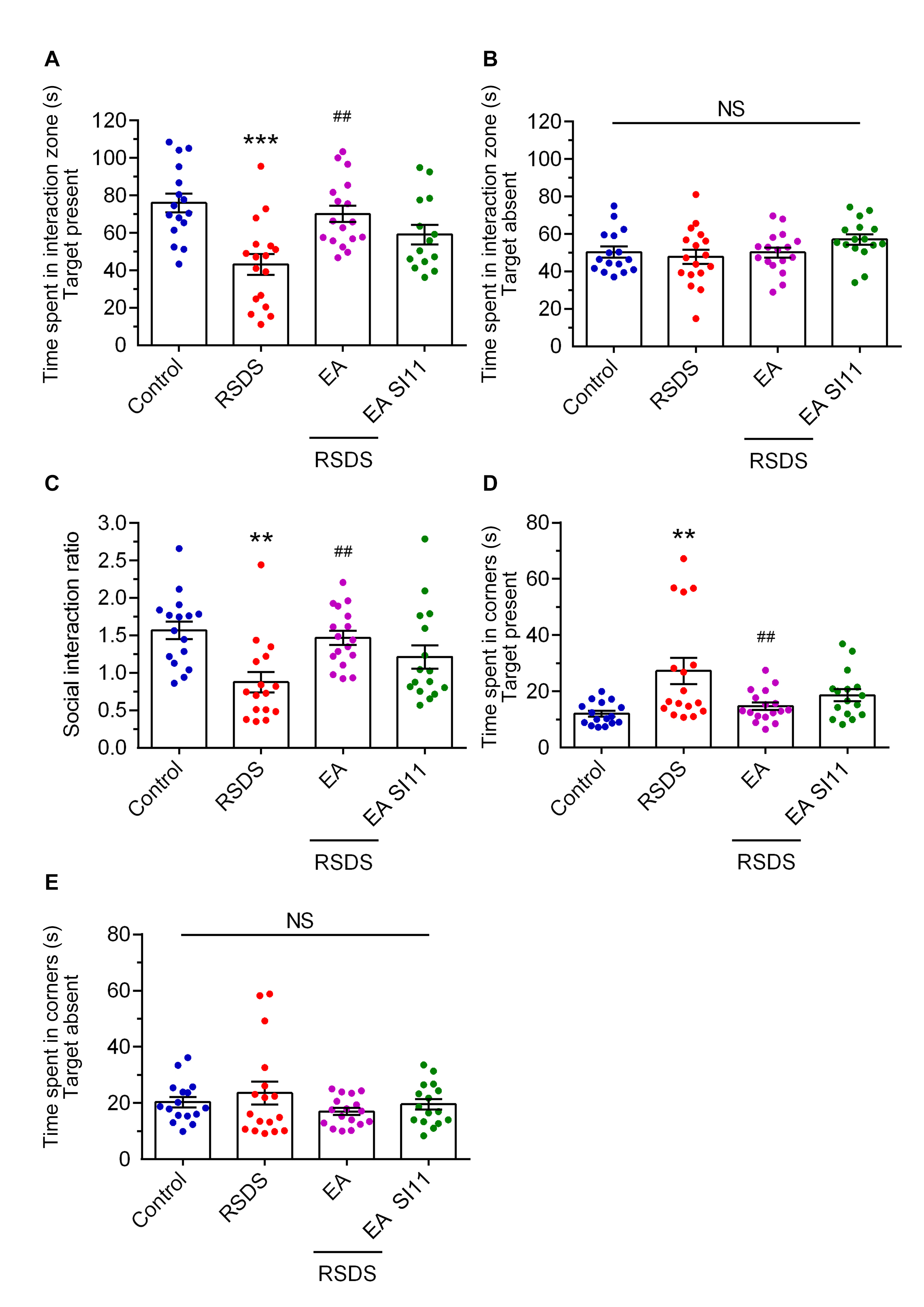

Supplement: Supplementary file 1 — Additional file 1: Fig. S1. Effects of electroacupuncture stimulation at the bilateral Tianzong (SI11) acupoints in the social interaction task. After RSDS, the experimental mice were introduced into the social interaction test apparatus. The time spent in the interaction zone was determined while the target was present (A) or absent (B). (C) The social interaction ratio was calculated by dividing the time spent in the interaction zone with the target present by the time spent in the interaction zone with the target absent. The time spent in the corners was determined by the interaction zone with the target (D) or without the target (E). Quantitative data are presented as the mean ± SEM (n = 5 each group). One-way ANOVA with a post-hoc Tukey’s test was used to examine the significance of the mean. ** p < 0.01 vs. the control group. *** p < 0.001 vs. the control group. ## p < 0.01 vs. the RSDS group. ANOVA: analysis of variance, EA: electroacupuncture, NS: not significant, RSDS, repeated social defeat stress, SEM, standard error of mean. [file 13041_2021_860_MOESM1_ESM.jpg]

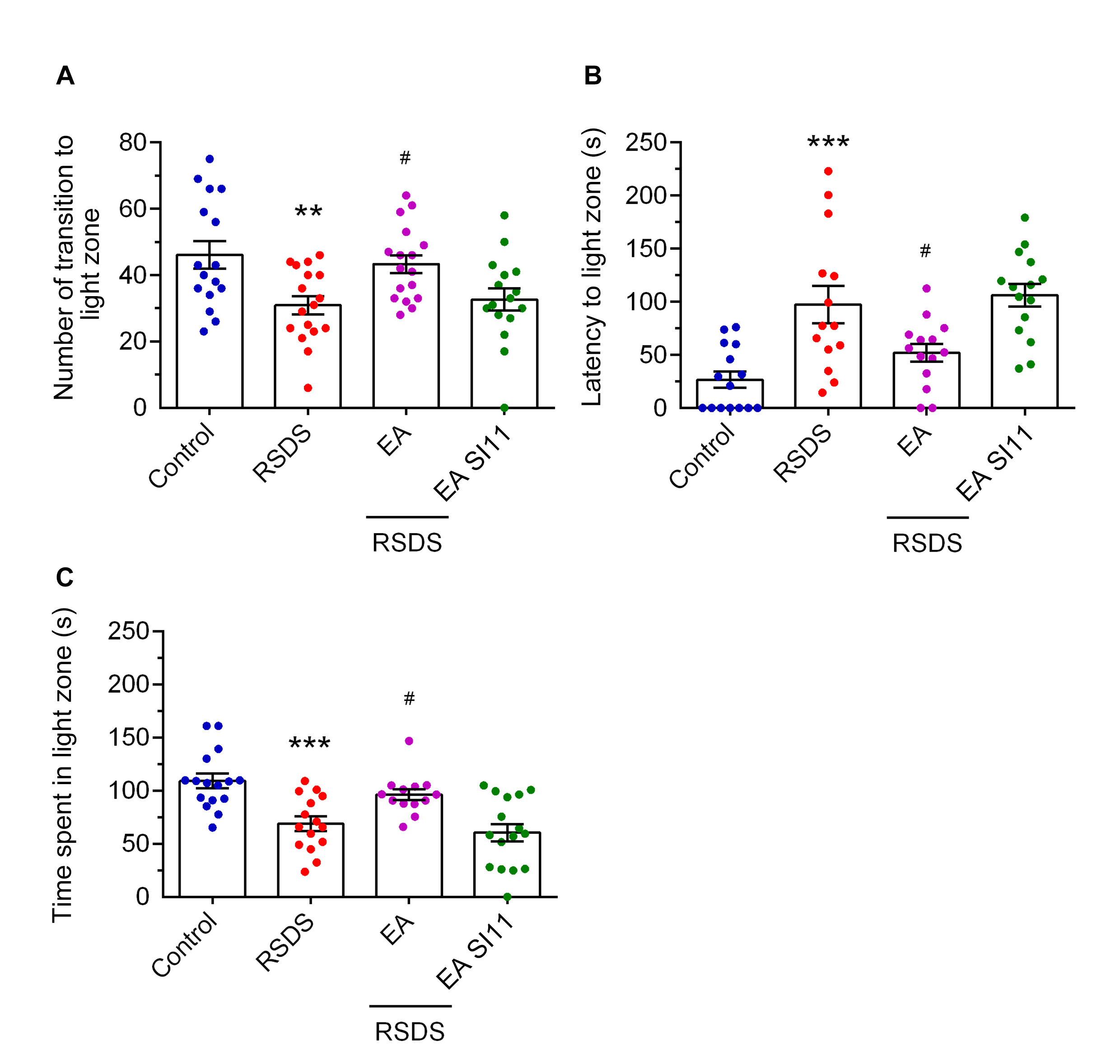

Supplement: Supplementary file 2 — Additional file 2: Fig. S2. Effects of EA stimulation at the bilateral Tianzong (SI11) acupoints in the light–dark task. After RSDS, the experimental mice were placed in a light–dark task apparatus. The frequency spent in the light compartment (A), the latency to the first light compartment (B), or the cumulative time in the light compartment (C) were determined. Quantitative data are presented as the mean ± SEM (n = 5 each group). One-way ANOVA with a post-hoc Tukey’s test was used to examine the significance of the mean. ** p < 0.01 vs. the control group. *** p < 0.001 vs. the control group. # p < 0.05 vs. the RSDS group. ANOVA: analysis of variance, EA: electroacupuncture, RSDS, repeated social defeat stress, SEM, standard error of mean [file 13041_2021_860_MOESM2_ESM.jpg]

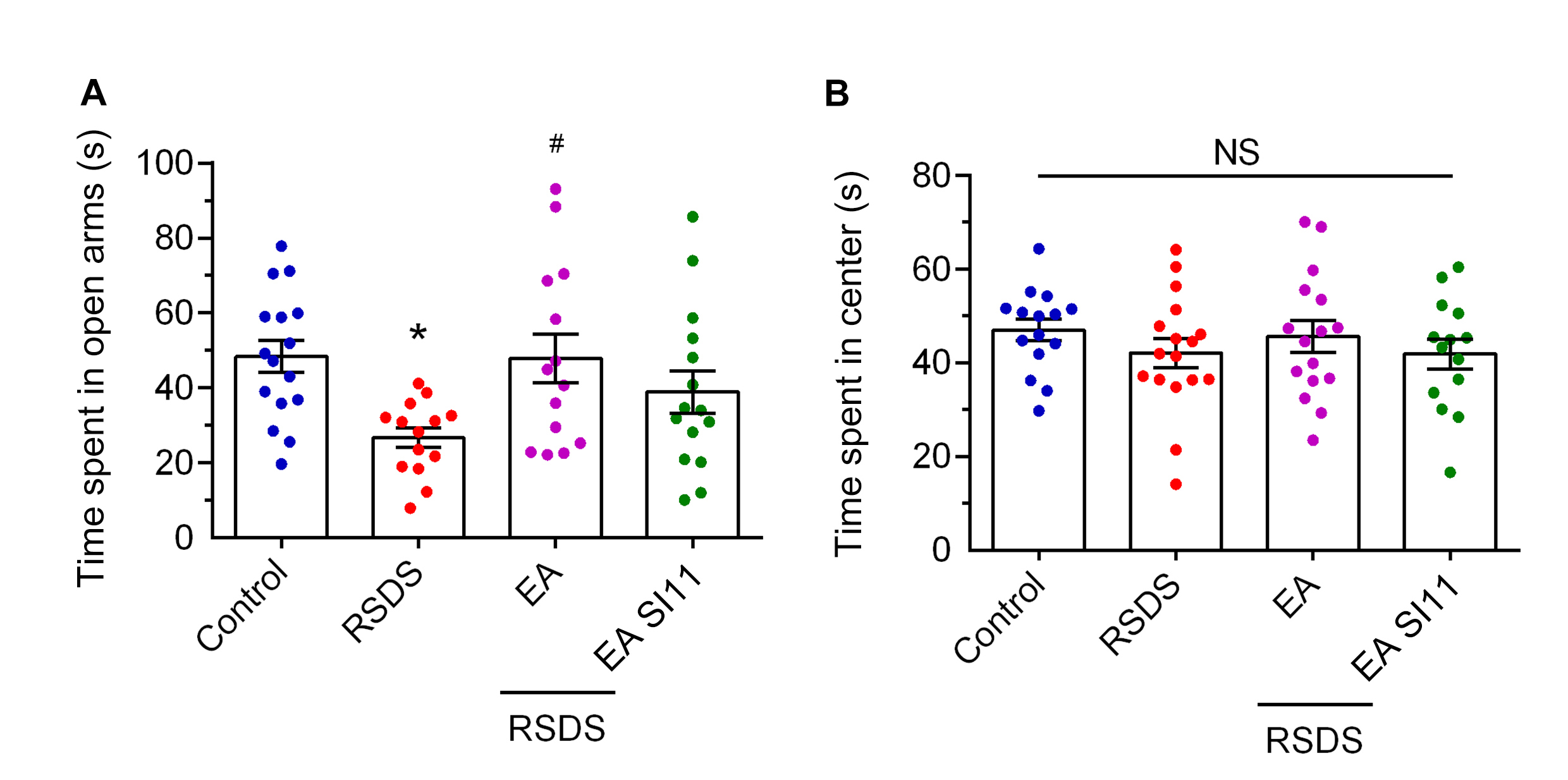

Supplement: Supplementary file 3 — Additional file 3: Fig. S3. Effects of EA stimulation at the bilateral Tianzong (SI11) acupoints in the EPM task. After RSDS, the experimental mice were introduced into the EPM apparatus. The time spent in the open arms (A) and center (B) of the apparatus were determined. Quantitative data are presented as the mean ± SEM (n = 5 each group). One-way ANOVA with a post-hoc Tukey’s test was used to examine the significance of the mean. * p < 0.05, RSDS group compared to the control group. # p < 0.05, EA group compared to the RSDS group. ANOVA: analysis of variance, EPM: elevated plus maze, EA: electroacupuncture, NS: not significant; RSDS, repeated social defeat stress. [file 13041_2021_860_MOESM3_ESM.jpg]

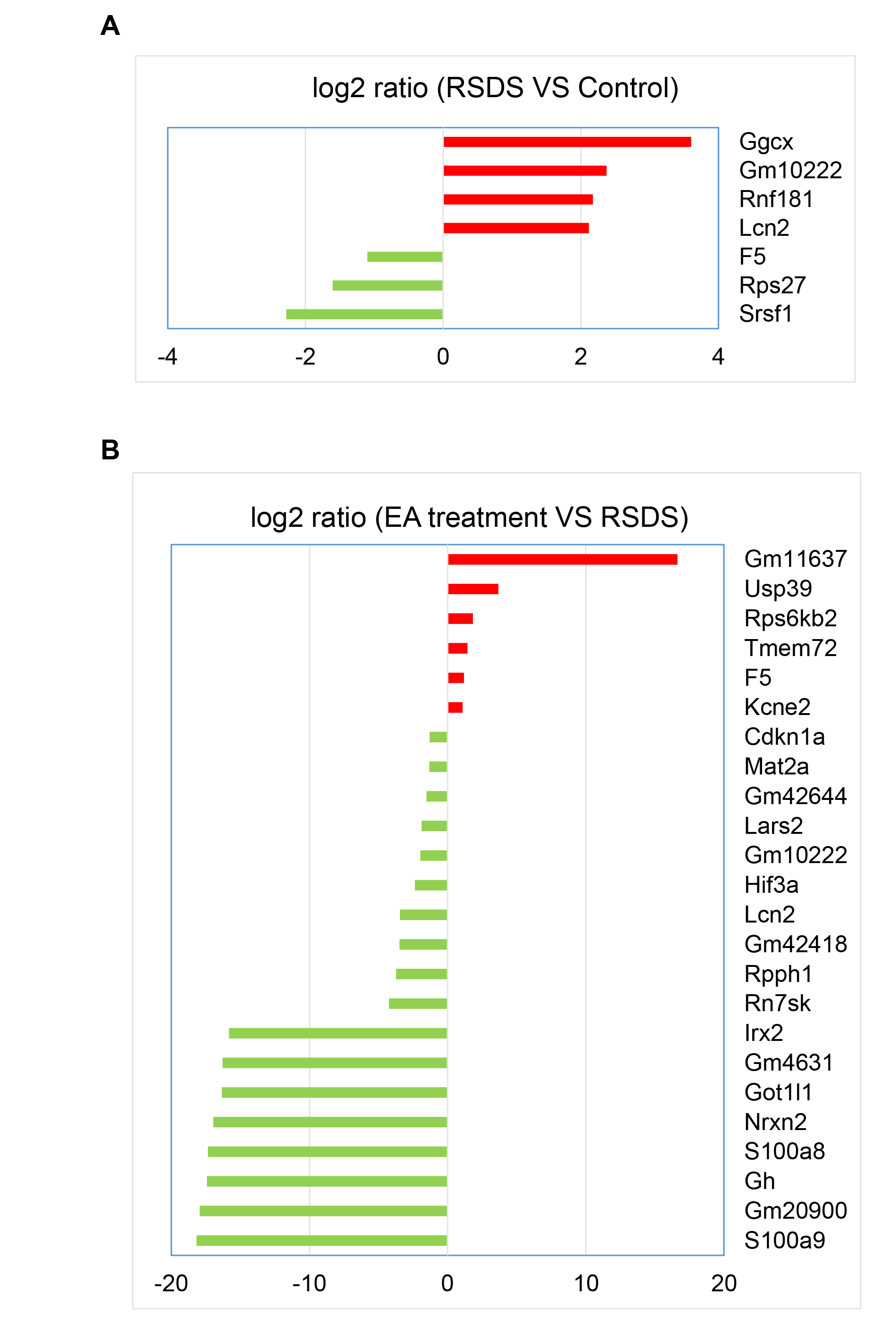

Supplement: Supplementary file 4 — Additional file 4: The bar chart of DEG expression. (A) The log2 ratio of DEG expression in RSDS VS Control. (B) The log2 ratio of DEG expression in EA treatment VS RSDS. [file 13041_2021_860_MOESM4_ESM.jpeg]

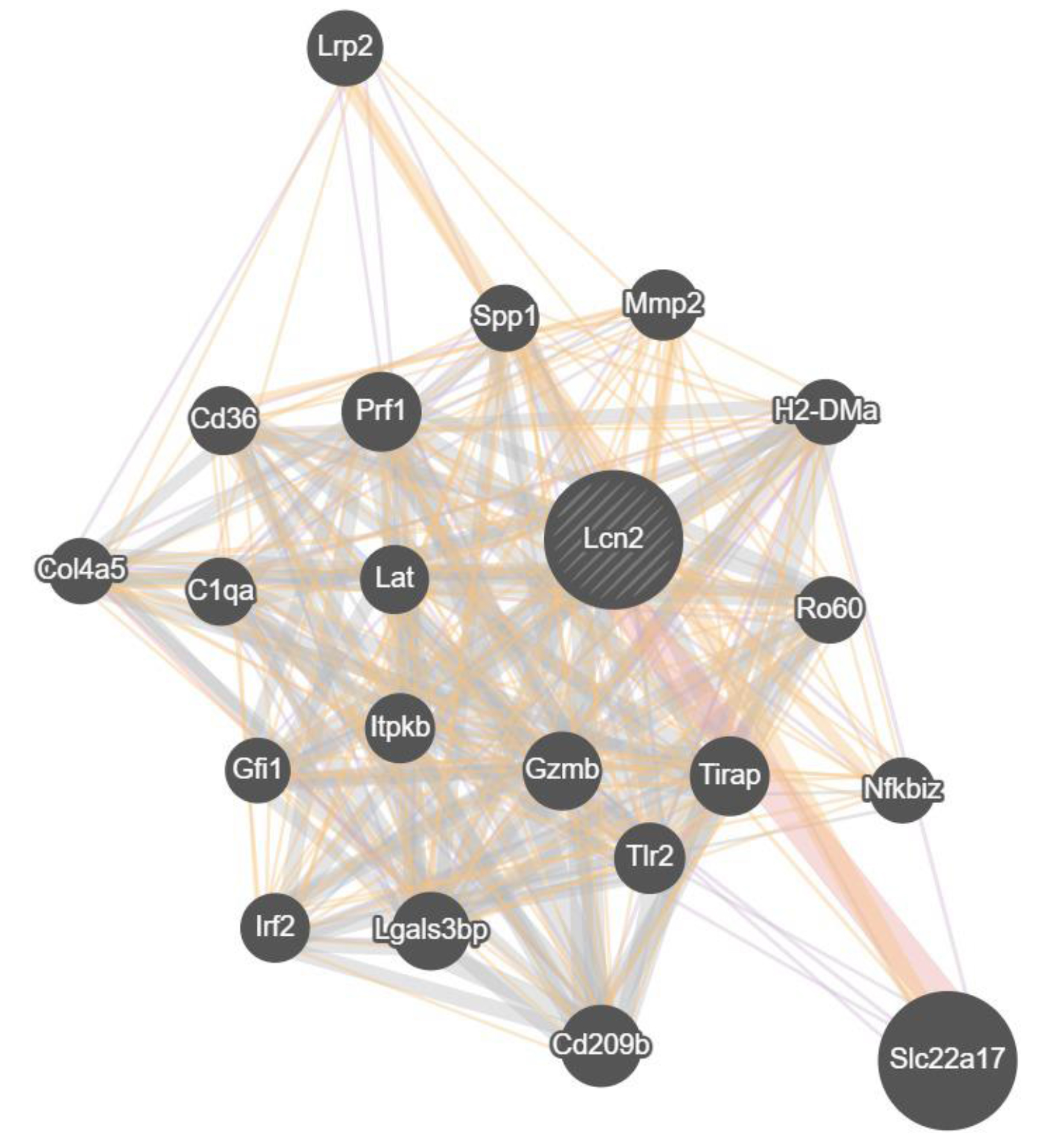

Supplement: Supplementary file 5 — Additional file 5: Protein–protein interaction network generated from GeneMANIA analysis. The size of each node presents the degree of connectivity in PPI network, and large node shares more connection to other nodes. [file 13041_2021_860_MOESM5_ESM.jpeg]

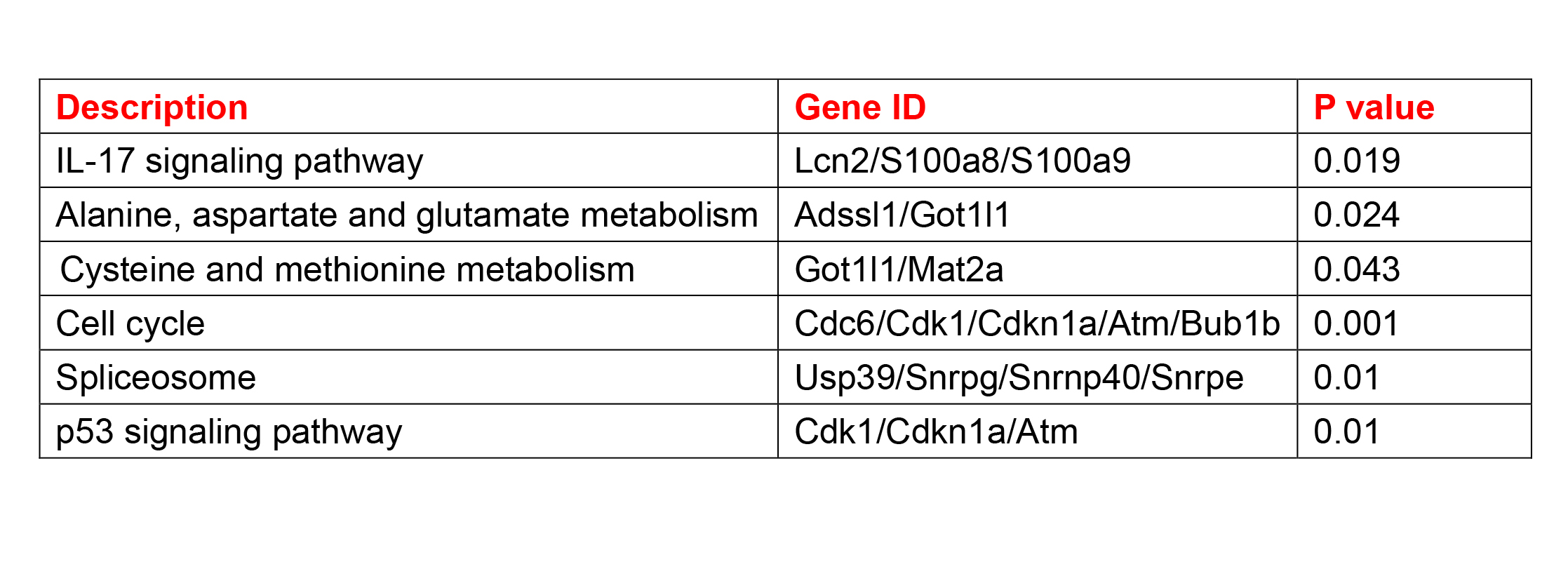

Supplement: Supplementary file 6 — Additional file 6: Top five pathways prediction of DEGs by KEGG. [file 13041_2021_860_MOESM6_ESM.jpeg]
